# Digital health interventions with healthcare information and self-management resources for young people with ADHD: *a mixed-methods systematic review and narrative synthesis*

**DOI:** 10.1007/s00787-025-02676-y

**Published:** 2025-03-01

**Authors:** Rebecca Gudka, Elleie McGlynn, Katherine Lister, Naomi Shaw, Emma Pitchforth, Faraz Mughal, Blandine French, John Headly Ward, Tamsin Newlove-Delgado, Anna Price

**Affiliations:** 1 University of Exeter, Exeter, UK; 2 Keele University, Newcastle-Under-Lyme, UK; 3 University of Nottingham, Nottingham, UK; 4 University of Cambridge, Cambridge, UK

**Keywords:** ADHD, Primary care, Digital health intervention, Systematic review, Young people

## Abstract

Attention deficit hyperactivity disorder (ADHD) is a common neurodevelopmental condition. Young people face challenges accessing ADHD healthcare in the UK, especially when transitioning between child and adult services. Digital health interventions (DHIs), as an adjunct to usual care, could improve access to healthcare information and condition-management support for this underserved group. This systematic review aimed to identify and synthesise evidence on DHIs suitable for young people aged 16–25 with ADHD, focussing on UK healthcare settings. We searched *MEDLINE*,* Embase*,* PsycINFO*,* IEE Xplore*,* ACM Digital Library*,* Cochrane Database of Systematic Reviews and Cochrane Central Register of Controlled Trials*,* Scopus*,* Web of Science Core Collection*,* ProQuest Dissertations & Theses Global* from inception to December 2023 for studies about DHIs for ADHD which provided healthcare and self-management information, and which included at least one participant aged 16–25 years. Studies were grouped by intervention type (healthcare & self-management information, symptom monitoring, and management tools), appraised using the Quality Assessment with Diverse Studies (QuADS) tool. A narrative synthesis was conducted. Nineteen papers representing 15 interventions and comprising 2651 participants were identified. Research studies spanned intervention development, feasibility/usability, pilots exploring preliminary effectiveness, and trials. QuADS scores ranged from 13 to 34 out of 39. Preliminary evidence of feasibility and acceptability was strongest for co-produced interventions. Limited evidence of effectiveness was found, except for psychoeducation DHIs which may be effective in reducing ADHD symptoms. Future research should build on this emerging evidence-base, emphasising co-produced DHIs tailored to young people with ADHD.

**Supplementary Information:**

The online version contains supplementary material available at 10.1007/s00787-025-02676-y.

## Introduction

### Background

Attention deficit hyperactivity disorder (ADHD) is a common neurodevelopmental condition characterised by inattention, disorganisation and/or hyperactivity and impulsivity, affecting 2–7% of children and adolescents, and 3% adults, worldwide [1–4]. Symptoms of ADHD persist into adulthood for up to 40% of young people [5]. ADHD can negatively affect an individual across several life domains, including physical and mental health, education, employment, relationships and mortality, with consequential economic burden on individuals, their families and supporters, and wider society [6].

Evidence shows that the negative outcomes associated with ADHD can be ameliorated with timely and appropriate treatment [7, 8]. This includes medication, which has been shown to have long- and short-term positive effects on ADHD outcomes, and non-pharmacological support, such as psychosocial interventions [9, 10]. A UK surveillance study estimated that between 270 and 599 per 100,000 people aged 17–19 years have an ongoing need for ADHD medication, and an even higher number require non-pharmacological support [11]. Alongside adequate treatment, National Institute for Health and Care Excellence guidance recommends that young people with ADHD who require ongoing ADHD support have a smooth transition between child and adult services [12]. During this vulnerable stage of life, withdrawal of treatment can have a particularly profound effect in young people, who are often undergoing multiple simultaneous transitions, such as those between educational settings, into new employment or moving away from a family home [13].

However, despite the importance of treatment, the Children and Adolescents with ADHD in Transition between Children’s and Adult Services study estimated that less than a quarter of young people who needed ADHD medication made the transition to adult mental health services [11]. Additionally, recent evidence from a national survey as part of the Managing ADHD services in Primary care (MAP) study found that 40% of commissioners reported waiting times of 2 or more years for patients to access adult ADHD services, with regional variation in the availability of these services [14]. Similar challenges to ADHD management exist in many European countries [15].

Limited access to specialist ADHD support, due to long waiting times, a lack of adult ADHD services, or because a person does not meet diagnostic criteria, increases pressure on primary care providers and may lead to suboptimal ADHD management [16–19]. Participants in the qualitative stream of the MAP study expressed concern over the limited resources available to support GPs in treating young patients with ADHD and systematic barriers to accessing support for ADHD via primary care, with characteristics of ADHD often exacerbating these challenges [20]. For example, due to difficulties with memory, attention or staying organised, booking appointments with a GP when required to call between certain hours can be difficult.

### Digital health interventions

Digital health interventions (DHIs) represent a rapidly evolving, promising opportunity to enhance ADHD healthcare provided within traditional NHS service structures (21). DHIs can improve attention and social function for people with ADHD and complement pharmacological treatment of ADHD [21, 22]. In addition, they may provide a cost- and resource-effective solution for young people on long waiting lists. At present, there is limited evidence for interventions which support young people with ADHD aged 16–25 [21, 23]. Despite the additional challenges this age group faces, a recent scoping review highlighted that they are often underrepresented in research and emphasised the importance of specific provisions tailored to this age range [24].

### Clinical relevance and significance

While UK guidelines recommend information provision and non-pharmacological interventions for people with ADHD [12], they are often not provided [20, 24]. DHIs could fill this gap in UK provision by supporting GPs (for example, providing interactive and targeted information at the point of need, such as screening tools and prescribing guidance), and supporting people with ADHD and their supporters (by providing information on care pathways and self-management tools, such as medication reminders). Developing DHIs requires systematic synthesis of existing evidence and must be guided by people with lived experience, healthcare professionals and service commissioners [25–27]. The current evidence and opportunity to implement DHIs present an urgent need for rigorous research to evaluate evidence-based digital healthcare solutions and inform future innovations, in line with the NHS Long Term Plan [28] and digitisation agenda.

### Aims of the current review

This systematic review aims to assess the quality of evidence for DHIs which provide healthcare information, education and self-management resources/strategies to young people (aged 16–25) with ADHD. It also aims to identify components which could be used (or adapted for use) in UK-based primary healthcare setting and evaluate their potential usability, acceptability, or efficacy in this context. The specific objectives of this systematic review are to:

- Identify and assess the quality of research evidence on the feasibility, usability, and/or effectiveness of DHIs suitable for use by young people with ADHD, to help them access healthcare, and support self-management of their condition.
- Identify and assess evidence on the potential usability of identified DHIs in a UK-based primary care setting.

## Methods

This review was written in accordance with the Preferred Reporting Items for Systematic Reviews and Meta-analysis (PRISMA) guidelines [29]. The review protocol was registered with the International Prospective Register of Systematic Reviews (PROSPERO) in August 2023 (CRD42023458822).

### Eligibility criteria

Included studies were published, peer-reviewed articles on DHIs for young people (aged 16–25) with ADHD. Both quantitative and qualitative papers were considered for inclusion.

#### Population

This review aimed to find interventions for young people aged 16–25 years. Studies were included if it was reasonable to assume that at least one participant was within the target age range. The assumption that at least one participant from any group was within the age range was determined by the upper and lower limits of mean age +/- standard deviation. The population of included studies were required to have ADHD, as defined by the authors of each study. Studies were included if the population had co-morbidities, and studies where the main population did not have ADHD were included so long as results for a subsample of the population with ADHD were presented separately.

Studies targeting parents of young people with ADHD were excluded unless the young person also participated.

#### Intervention

Studies investigating DHIs aimed at informing, educating and providing self-management resources/strategies for young people were included. Relevant healthcare information could include details of local care pathways or medication, living with ADHD as a long-term condition or experiences and stories from other people with the condition. DHIs for management or self-management for ADHD could include medication monitoring and reminders, psychoeducation, and DHIs which aim to reduce symptoms of common comorbidities such as mental health conditions or substance use.

Non-interactive healthcare resources and resources without a focus on health, for example, educational aids or classroom management, were excluded. Interventions which aimed to improve core symptoms of ADHD through neurocognitive training of neurological components such as reaction time, processing speed and visual-motor control, without any self-management or informative element, were excluded. Similarly, neuro- and bio-feedback interventions were excluded unless they explicitly stated that they include healthcare information or self-management components. To be deliverable in a UK primary healthcare setting, interventions which required clinician engagement for individual patients were excluded.

#### Outcomes and comparators

Studies which use any measure of effectiveness, acceptability, or participant engagement were included. Qualitative studies were included where participants are asked about their experiences of an intervention. Provided the intervention is designed for use by 16–25-year-olds, the outcome measures may be reported by any stakeholder, including young people, their supporters, or clinicians.

All comparators were considered, including usual care. Due to the anticipated methodological heterogeneity, we considered studies that used no comparator group.

Study protocols and development workshops/studies were excluded unless the development study produced a prototype intervention which was tested with stakeholders. Forward citation searching of protocols and development studies was performed to check whether results had been published since publication of the protocol.

#### Additional criteria

Studies had to be reported in English, as time and resource constraints did not allow for translation of non-English texts. Included records had to be conducted within any high-income countries as defined by the World Bank list to ensure they were relevant to a UK healthcare setting [30]. Included articles had to be published since the introduction of the Health and Social Care Act in 2008, as the introduction of the Act resulted in changes to services for patients, and due to the rapidly evolving nature of digital technologies it is unlikely that DHIs developed prior to this would remain relevant to. Grey literature and conference abstracts were excluded. For a summary of the eligibility criteria of papers, see Table 1.

**Table 1:** Summary of eligibility criteria for included studies

Key criteria	Additional notes
Young people (aged 16 to 25 years) (Population)	Include studies where it is reasonable to assume (considering the mean age and standard deviation) that at least one of the participants is within the target age range (16–25 years). Include studies where a young person *and* supporter are both included in sample. Exclude studies where supporter alone is included in the sample.
Study focussed on people with ADHD (Population)	
Digital health interventions (DHIs) (Intervention)	Digital health interventions defined as: • Health apps or software • Interactive tools (including websites where interactive elements are present) • Automated SMS or digital messaging • Exclude if SMS or digital messaging is *not* automated or requires clinician/administrator engagement beyond what would be deliverable in the context of UK primary healthcare
DHIs for people with ADHD with: • Relevant healthcare information (e.g. care pathways/information/anecdotes about ADHD) • Support management and self-management (e.g. meds reminders, psychoeducation, support accessing services) (Intervention)	Include neuro/bio-feedback interventions *only* if the desired outcome is related to management or self-management (for example, exclude if neurofeedback is for memory training only) Exclude if educational or classroom aids are designed with educational attainment outcomes in mind Exclude if diagnostic or screening assessment tools
Any measure of effectiveness, acceptability, usability, or participant engagement with or experiences of the DHI (Outcome)	
Peer-reviewed, primary research, English language (Study features)	Exclude systematic reviews, commentaries, letters to editor (non-exhaustive)

### Search strategy

The search strategy, adapted from previous work in consultation with an information specialist (NS) [24], combined free-text terms and subject headings for ADHD, apps, and digital information sources. Intervention terms were derived from NICE MEDLINE and EMBASE search filters and supplemented with additional terms for digital health information sources [31]. Databases (MEDLINE, Embase, PsycINFO (Ovid), IEE Xplore, ACM Digital Library, Cochrane Database of Systematic Reviews (CDSR) and the Cochrane Central Register of Controlled Trials (CENTRAL) (Wiley), Scopus (Elsevier), Web of Science Core Collection (Clarivate), ProQuest Dissertations & Theses Global) were searched from inception without limitation on date, language, or publication type. No filters/terms related to the age range were applied to avoid missing any potentially relevant records. Initial searches were conducted on 28 July 2023 and updated on 17 December 2023. Additionally, RG carried out forward and backward citation searching on all included full-text papers and relevant systematic reviews on 7 February 2024 using Citation Chaser [32]. Full search strategies are available in Appendix 1.

### Study selection

Following searches, results were deduplicated in EndNote (Version 20) (Clarivate). Four authors (RG, EM, KL and AP) screened all records. All titles and abstracts derived from the original and update searches were dual screened against eligibility criteria by two independent researchers. 10% of records retrieved from citation searching were dual screened, with the remaining screened by RG alone. Full reports were retrieved and screened by two independent researchers to determine a final list of studies which met inclusion criteria. Throughout screening stages, inconsistencies were discussed and resolved through individual discussions and weekly team meetings. Cadima was used to manage screening and selection processes [33]. Seven full text articles were not available despite requests to the authors.

### Data extraction & synthesis

Two reviewers (RG and AP) extracted data from included studies using a tool developed from the findings of a recent scoping review and principles from the Cochrane Handbook [24, 34]. The data extraction tool was piloted and refined by RG and AP, who piloted three studies each using the final tool. Data extraction for all studies was checked by a second reviewer (EM and KL). Discrepancies were discussed with the whole team until consensus was reached.

The key outcomes for the review were intervention descriptions and component parts, narrative descriptions of findings related to ADHD outcomes, other health and wellbeing outcomes, and accessibility, usability and feasibility. Key characteristics of the study (aims, methods, sample characteristics) were also extracted. Published protocols were consulted in the case of missing or not reported information, otherwise data was extracted as “not reported” and this was considered in the quality appraisal.

Due to the expected heterogeneity of study design, meta-analysis was not planned, and narrative synthesis was conducted, with a systematic approach to the tabulation and grouping of studies, and to the descriptions of preliminary effect, feasibility and implementation of interventions [35]. Synthesis was grouped by intervention type, based on the primary components of the intervention.

### Study quality

Due to the high proportion of feasibility and pilot studies and heterogenous study designs, separating the process of quality assessment between study designs, as protocolised, was not optimal. Thus, following consultation with an evidence synthesis specialist, the Quality Assessment with Diverse Studies (QuADS) tool was deemed the most appropriate quality appraisal tool [36]. Two reviewers (RG and AP) undertook dual quality appraisal of included studies, with disagreements resolved through discussion.

## Results

### Description of study selection and included studies

**Fig. 1:**
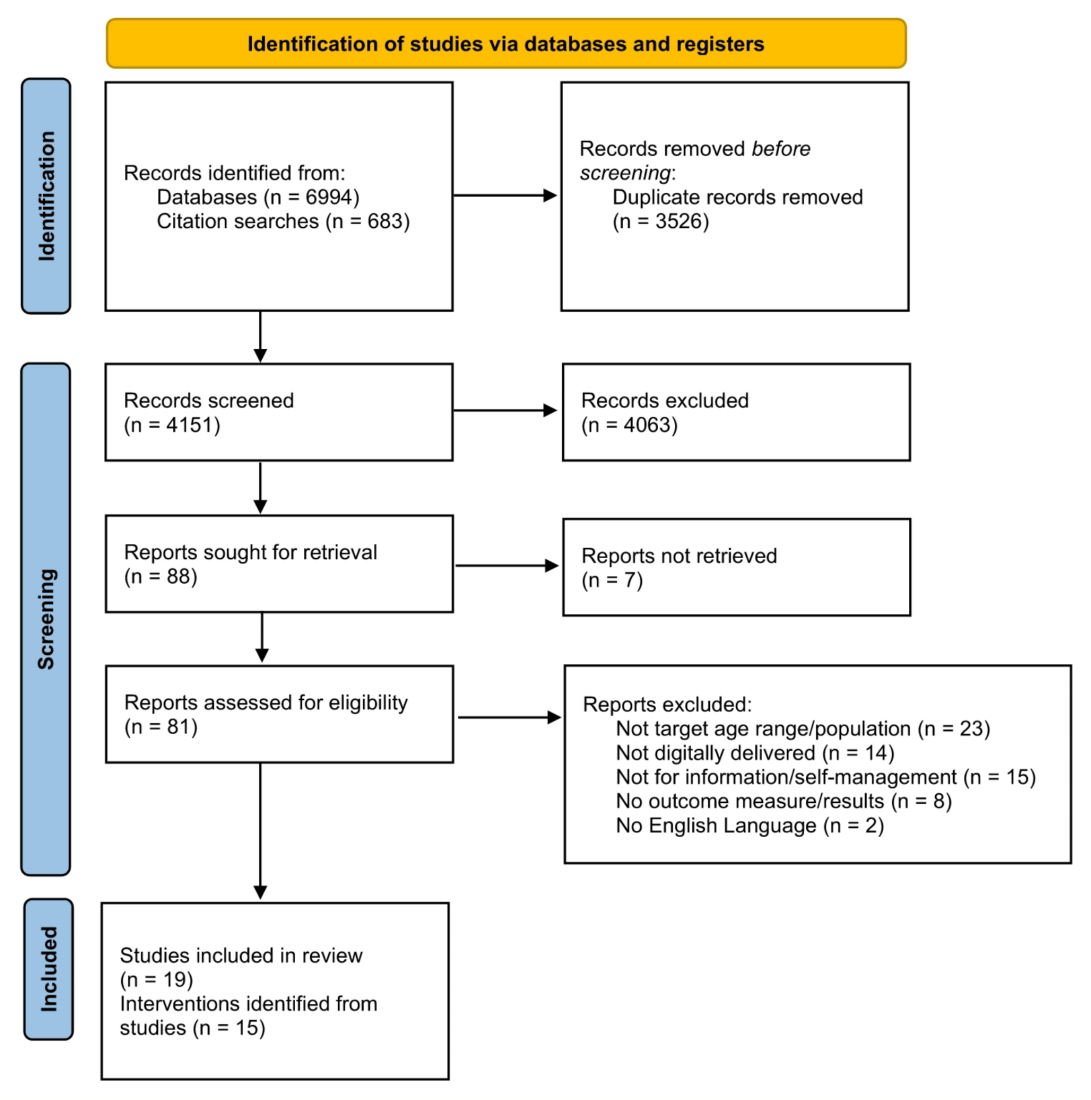
PRISMA flow diagram

Figure 1 shows the PRISMA flow diagram of search and screening results. The searches identified 6994 records, plus an additional 683 identified through forwards and backwards citation searches of included studies and relevant systematic reviews. After de-duplication, 4151 records were screened. Of these, 4063 were excluded because they did not meet inclusion criteria. We obtained 81 full-text articles to assess for eligibility, of which 62 were excluded (see reasons for exclusion in Appendix 3).

**Table 2:** Study characteristics, grouped by primary intervention type, and listed in order of evidence type [e.g., from trials to initial development)

Author (year, country)– Intervention name*	Study type (pilot, feasibility, etc.)	Sample size	Age range in years (mean age (SD))	% Female	Ethnicity	Control/comparator group
Psychoeducation**						
Kenter (2023, Norway)– **MyADHD (37)**	RCT– parallel (2-arm)	110 (Intervention = 61, Control = 59)	20–77 (40.9 (10.6)) (Intervention = 40.1 (10.0). Control = 41.2 (11.2))	80	NR	Text-based psychoeducation module
Nordby (2022, Norway)– **MyADHD (38)**	Multiple randomised trial– parallel (2-arm)	109	22–62 (36.1 (9.1))	80.7	NR	None
Kenter (2022, Norway)– **MyADHD (39)**	Usability– think-aloud & usability evaluation	5	25–62 (38.4 (16.3))	40	NR	None
Flobak (2021, Norway)– **MyADHD (40)**	Participatory design & evaluation,	109	22–62 (36.1 (9.1))	80.7	NR	None
Nasri (2023, Sweden)– **iCBT (41)**	RCT– parallel (3-arm)	104 (Intervention = 26, Active control = 27, TAU = 31)	Intervention = 36.7 (11.4), Active control = 35.897 (9.4), TAU = 37.2 (10.3)	69.2	NR	Active control = applied relaxation therapy. Inactive control = TAU
Petterson (2017, Sweden)– **InFocus (42)**	RCT– parallel (3-arm)	45 (Intervention = 13, Active control = 14, Waitlist group = 18)	Intervention = 38.92 (8.50), Active control = 39.64 (12.44), Waitlist group = 33.78 (10.07)	54.8	NR	Active control = internet-based CBT with weekly group sessions. Inactive control = waitlist group
Ahlers (2022, Switzerland) -**CANReduce (43)**	RCT (subgroup analysis)– parallel (3-arm)	367 (With ADHD = 94, Without ADHD = 273)	NR (27.9 (7.5))	28.3	NR	Comparator group without ADHD
Selasowski (2023, Germany)– Chatbot* (44)	RCT– parallel (2-arm)	34 (Chatbot = 17, Control = 17)	Chatbot = 19–44 (29.6 (7.6)), Control = 20–52 (29.7 (9.5))	52.9	NR	Self-guided app-based psychoeducation
Knouse (2022, USA)– **InFlow (45)**	Feasibility & usability– Preliminary effect	240	18–46 (29.15 (7.14))	69.2	Caucasian (78.8%) African American (9.2%) Native American (4.2%) Asian/ Pacific Islander (5.8%) Other (9.6%)	None
Jang (2021, Korea)– **Tokadi (46)**	Pilot (feasibility & usability)– Preliminary effect	46 (Chatbot = 23, Control = 23)	NR (Chatbot = 26.7 (8.97), Control = 22.87 (5.44))	57	NR	Paperback self-help book
Shelton (2022, USA)– IBI* (47)	Feasibility & acceptability of *concept*	235 (Minimal = 68, Full = 68, Tailored = 99)	18–35 (27.54 (4.29)) (Minimal = 26.72 (4.48), Full = 27.75 (3.94), Tailored = 27.97 (4.36))	54.9	White (76.2%) Mixed (6.8%) Black (6%) Asian (5.1%) Hispanic (3.4%) Other (2.5%)	Same intervention presented in 3 conditions (Full condition, tailored condition, minimal condition)
Symptom monitoring**						
Surman (2022, USA)–Treatment Optimisation* (48)	Pilot (usability & utility)	206	NR (37.3 (13.1))	58.7	Caucasian (76.7%) Asian (3.9%) Black/African American (0.5%) Multiple races (2.4%) NR (16.5%)	None
Kennedy (2022, USA)– **Ecological Momentary Assessment (49)**	Preliminary effectiveness study	90	13–18 (14.7 (NR))	34	Caucasian (76.7%) Black/African American (13.3%) Asian (8.9%) Hispanic (3.3%)	None
Leikauf (2019, USA)– **StopWatch (50)**	Pilot (feasibility)– Preliminary effect	32	8–17 (11*) *Median	47	NR	None
Schoenfelder (2017, USA)– **FitBit Flex (51)**	Pilot (feasibility)– Preliminary effect	11	14–18 (15.5 (1.4))	54	Caucasian (80%) Asian American (10%) Multi-racial (10%)	None
Practical interventions**						
Biederman (2019, USA)–Medication Reminders* (52)	Preliminary effectiveness study	552 (Intervention = 92, TAU = 460)	Intervention = NR (32.7 (9.8)) TAU = NR (31.5 (7.7))	39	Intervention = Caucasian (82%) TAU = Caucasian (92%)	Treatment as usual
Biederman (2020, USA)–Medication Reminders* (52)	Pilot (effectiveness & acceptability)– Preliminary effect in primary care setting	448 (Intervention = 112, TAU = 336)	Intervention = NR (35.9 (10.0)) TAU = NR (33.7 (7.0))	55	Intervention = Caucasian (86%) TAU = Caucasian (86%)	Treatment as usual
Healthcare and self-management information**						
Wright (2023, USA)– **IUEVO (53)**	Development (co-design)	11 (participants with ADHD = 5)	11–17 (NR (NR))	63.63	White (81.81%) Black/African American (18.18%) Hispanic/Latino (9.09%)	None
Luiu (2018, Switzerland)– Luiu* (54)	Feasibility– qualitative evaluation	6	20–55 (NR (NR))	NR	NR	NR

*Where name not provided, a descriptive acronym (or lead author name) has been used

**Primary aim/focus of the intervention, as described by study authors

TAU = Treatment as usual, NR = Not reported, CBT = Cognitive behavioural therapy, RCT = Randomised controlled trial

One study (Rachamim 2021) was identified, as a potential include, but without sufficient reported information to be sure [37]. During data extraction, further investigation via a linked article [38] clarified that this intervention had not been designed for an ADHD population, therefore this study was excluded.

19 studies, which investigated 15 interventions of various modality and content type were included in the review. Table 2 describes the characteristics of each included study; Table 3 provides an overview of each intervention, evidence type, intervention components, summarised findings, and quality appraisal. More detailed descriptions of interventions and the relevant outcome domains can be found in Appendix 4. In addition to the 19 records which have been included in this study, two conference articles and three PhD theses were identified as relevant during the screening process. They met all the criteria except for being peer-reviewed publications, and are included in tables in Appendix 2.

### Study design

Five studies were randomised controlled trials (RCT) [39–43]. One study used a multiple randomised trial design without a control group [44]. Two explored the development processes of the intervention [45, 46]. Eight pilot, feasibility or usability studies which collected preliminary effect data were included [47–54]. Three studies were feasibility or usability studies evaluating the intervention concept without collecting preliminary effectiveness data [39, 55, 56]. Of the 19 studies included, only nine used control or comparator groups.

### Participants

Across included studies, there were 2651 participants. The youngest participant was eight, and the oldest was 77 years old. As expected from our previous scoping review [24], no studies focussed exclusively on people aged 16–25 years-old. While this reduces the specificity of the findings presented here, results still hold relevance for this age group, as all studies included at least one participant within the desired age range. The proportion of female participants ranged from 28.3 to 80% (mean proportion of female participants = 55.2%). This is unexpected, as females have previously been underrepresented in ADHD research, and studies on ADHD prevalence often find that males are more likely to have ADHD than females [1, 2]. Although, this has been attributed to referral bias [2]. The lack of non-white participants in studies is reflective of literature which shows that white individuals are often overrepresented in ADHD research, despite little evidence of associations between ADHD prevalence and ethnicity [57, 58]. This systematic review aims to explore interventions which *could* be deliverable in a UK healthcare setting. Thus, it is important to note that none of the included studies were carried out in the UK. Nine were carried out in the USA, four in Norway, two in Switzerland, two in Sweden, one in Korea and one in Germany. In the eight studies which reported on ethnic breakdown of samples, the range of white/Caucasian participants was 76.2 − 86%.

### Quality of included studies

The quality scores across included studies, which comprised 13 categories scored 0–3, ranged from 13 to 34 (Median score = 28) out of a total possible score of 39. Scores closer to 39 indicate higher quality. While Harrison et al. [36] state that cut-off scores for considering studies to be high or low quality would be arbitrary, following discussion with the research team, and to provide clarity for readers, we have colour coded quality appraisal scores to reflect where studies scored across the range of possible scores, see Tables 3 and 4. The decision to colour code is in line with the research team’s assessment of the quality of the evidence, as informed by each criterion of the framework, their importance in the context of the aims of this review, and a discussion of the specific strengths and limitations of each included study. Included studies were strongest on statement of research aims (M 2.6) and generally showed weakest performance in terms of evidence of stakeholder involvement (M 0.8), appropriate sampling to address research aims (M 1.9), and justification for choice of analytic method (M 1.9). Only three (16%) studies scored a 2 or above on evidence of stakeholder involvement [45, 46, 59]. Table 4 provides a breakdown of scores for all domains on the QuADS tool.

**Table 3:**
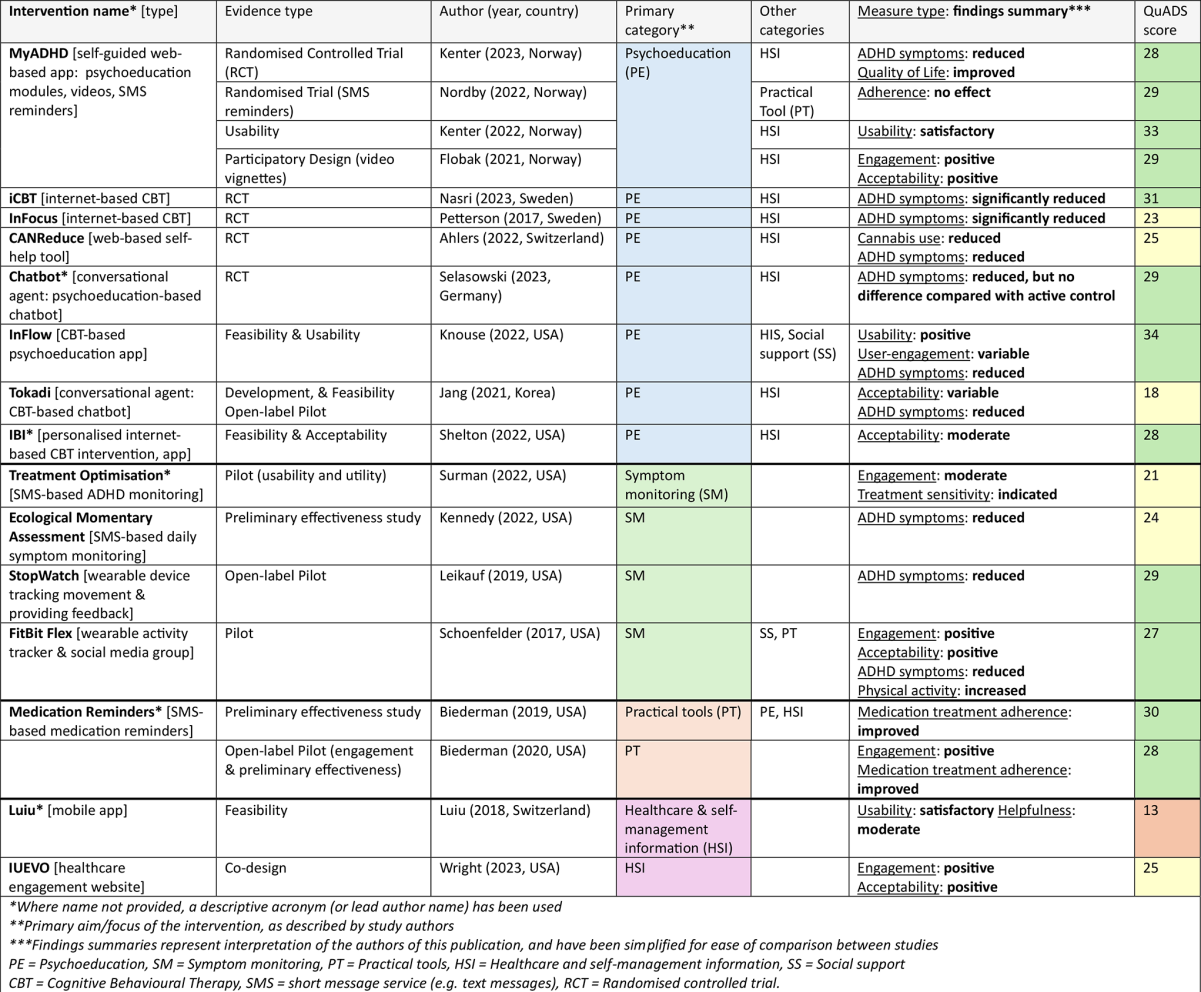
Overview of included studies grouped by primary category, and ordered by evidence type

**Table 4:**
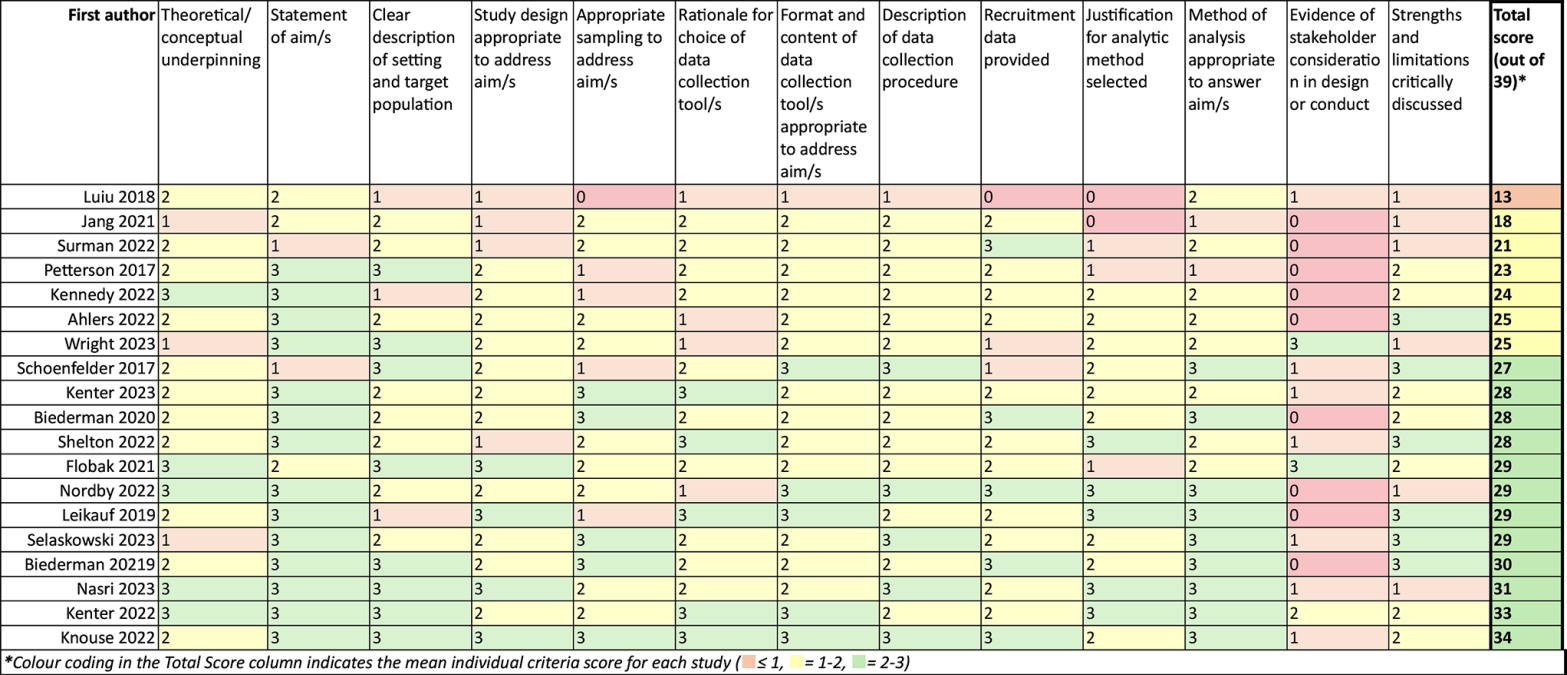
Full quality appraisal scores using the quads criteria, listed in order of lowest to highest quality studies

### Narrative synthesis

**Fig. 2:**
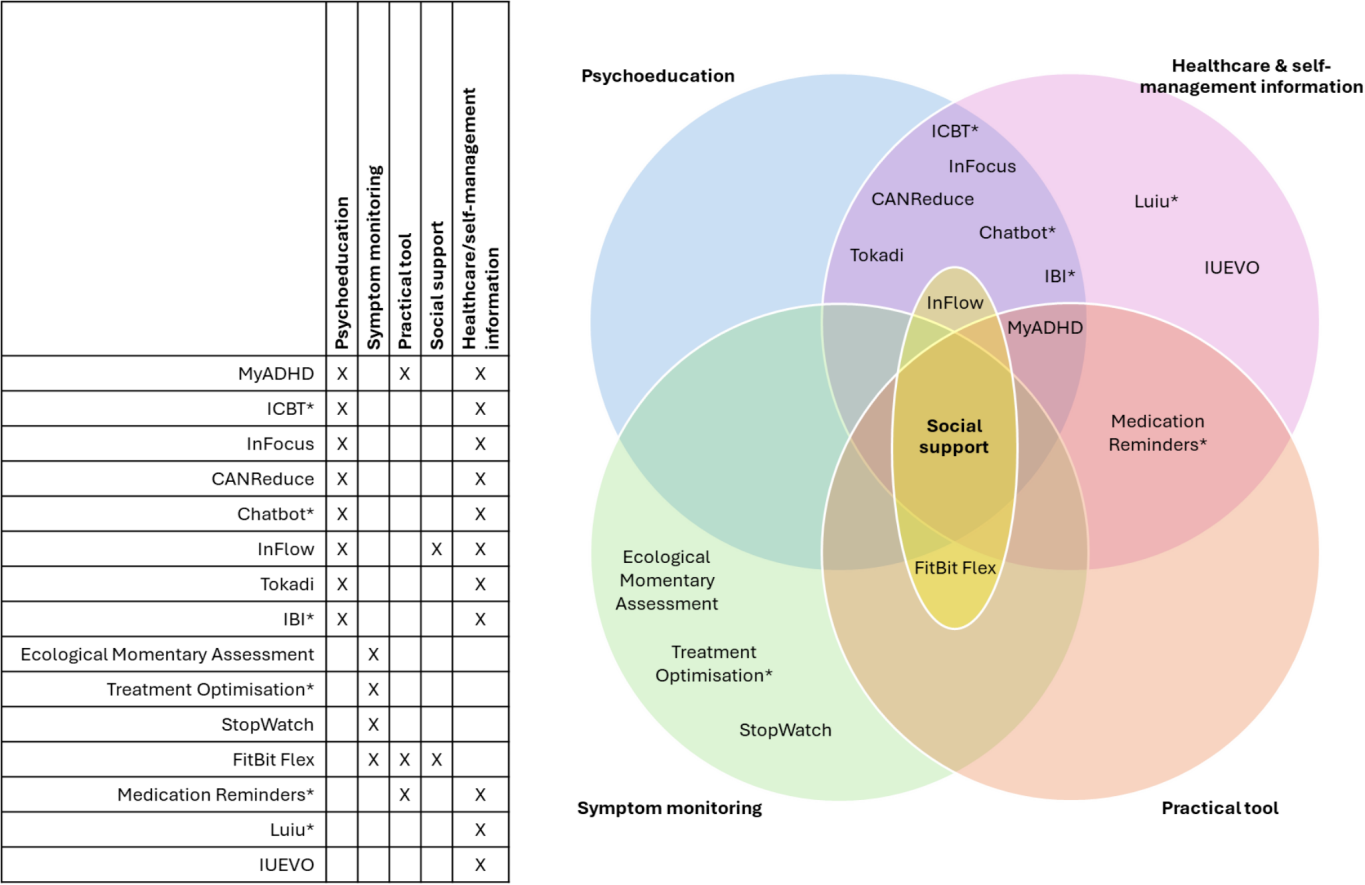
Table and Venn Diagram of included interventions, illustrating overlapping intervention component types

The included studies were reviewed in depth and grouped by intervention type and content delivered, then ordered to reflect the hierarchy of evidence [60]: from trials to pilots, to development research (RG & AP). For interventions spanning multiple categories (see Fig. 2), the primary category was used. Categories were shaped by current definitions in the literature [61–63], an in-depth review of the aims and interventions of each included study. Full detail of the category descriptions is available in Appendix 5. The final four categories were:

- **Psychoeducation** for people with ADHD involves delivering a validated therapeutic manual (E.g., Cognitive Behavioural Therapy) which may be adapted for ADHD [64, 65]. It includes information and empowering training for patients to promote awareness, and provides tools to manage, cope and live with ADHD, and promote behaviour change.
- **Symptom monitoring** interventions involve collecting data through self-reports or devices such as smartwatches to track patients’ symptoms, often providing patients with visual summaries of the data.
- **Practical interventions** vary in their nature but generally facilitate self-management strategies by providing stand-alone templates, prompts or activities which may help to alleviate levels of impairment.
- Finally, **healthcare & self-management information** is the least intensive intervention type, and generally provides information about ADHD, healthcare, self-management strategies and signposting to other resources, provide tools, or encourage behaviour change without following therapeutic manuals.

### Psychoeducation

Eight interventions (11 studies) provided psychoeducation, making it the most common intervention type. All interventions had modules which comprised ADHD-specific healthcare & self-management information [39–45, 47, 48, 55, 59], and **InFlow** also provided social support by facilitating participant interaction with a community of other app users [47]. **MyADHD** was evaluated with the additional practical tool of SMS reminders [44]. Psychoeducational interventions had the highest level of evidence of all categories, with five RCTs, one multiple randomised trial, five development or feasibility studies, and eight of 11 studies receiving an average score of 2 + for each QuADS scoring domain (see Table 3).

The therapeutic techniques varied: **InFlow**, **Tokadi**, Internet-based intervention (**IBI**), and **InFocus** [41, 47, 48, 55] used Cognitive Behavioural Therapy (CBT). **CANReduce** combined motivational interviewing with CBT [42]. **MyADHD** used CBT combined with dialectical behavioural therapy (DBT) and goal management training (GMT) [39, 44, 45, 59]. Internet-based CBT (**iCBT**) by Nasri et al. used CBT and DBT combined [40]. Finally, Selaskowski et al. used a previously validated manual to form the basis of their **Chatbot** intervention [43, 66].

Outcome measures for ADHD symptoms included the Adult ADHD Self Report Scale v1.1 Symptom Checklist (ASRS) [39, 40, 42], Barkley Adult ADHD Rating Scale—IV and Barkley Functional Impairment Scale [47], Conners’ Adult ADHD Rating Scale [48], Integrated Diagnosis of Adult ADHD, revised version and ADHD self-report scale [43], and the Current Symptoms Scale -Self-report form [41]. Other outcomes measured include quality of life, other health-related items such as depression and anxiety, and feasibility/acceptability data collected via interview questions or app/intervention usage data. **CANReduce** aimed to reduce the number of days participants consumed cannabis, measured using the Cannabis Use Disorder Identification Test and the Severity of Dependence Scale [42].

Five RCTs evaluated intervention effectiveness against an active control [39–41, 43] or in a non-ADHD population [42]. Findings from RCTs suggest that digitally delivered psychoeducation reduced ADHD symptoms significantly more than treatment as usual (TAU) or no treatment. Reported changes in ADHD symptoms varied from 4.65 to 8.70 when self-rated using the ASRS, and all RCTs found clinically significant improvements [39–43]. **iCBT** and internet-based applied relaxation training (iART) both reduced ADHD symptoms more than the TAU condition, and to the same extent [40]. However, **iCBT** was superior to TAU in reducing symptom severity of depression, perceived stress, functional impairment and life satisfaction. In contrast, iART was not, suggesting that **iCBT** may be more effective at reducing total ADHD and co-morbid symptoms. Compared to the face-to-face version of the same treatment manual, **iCBT** showed smaller effect sizes when delivered digitally. However, recruitment challenges prevented the study reaching its targets, leaving it underpowered. Petterson et al. found that **InFocus**-self-help format was superior to no treatment, whereas the group-delivered format was not [41]. **CANReduce** effectively reduced cannabis consumption and improved ADHD symptoms. Researchers deemed it helpful for people with ADHD because, despite a high early dropout rate, they adhered to the treatment at the same rate as those without ADHD [42].

**MyADHD** showed large effect-size improvements in self-reported ADHD symptoms and quality of life post-intervention and at 3-month follow-up in an RCT [39]. The usability study showed an average usability score of 77.5 out of 100 following iterations based on the first round of feedback [59]. A multiple randomised trial of **MyADHD** found that receiving SMS reminders to complete the intervention significantly affect module completion, frequency of logins or coping strategy practice [44]. Despite this, participants completed an average of 4.6 out of six modules, and 56% of participants were considered to have completed the intervention [44]. Selaskowski et al. found that their **Chatbot** supported psychoeducation intervention led to a decrease in observer- and self-rated ADHD symptoms but was also no more effective than a self-guided app-based intervention [43]. While the finding was not significant, self-rated ADHD symptoms were reduced to a greater extent in the self-guided group than in the chatbot supported group.

**Tokadi**, another chatbot-supported psychoeducation intervention, outperformed a non-digital self-guided book on ADHD-self-management, with open-label pilot findings of reduced self-reported ADHD symptoms [48]. Participants reported liking the empathetic mascot, alarm, tracking features and ease of accessibility. However, participants found the chatbot conversation flow unnatural and regarded unintuitive user-interfaces as a negative feature. A feasibility/usability study found that more time spent using the app was associated with greater reductions in ADHD scores [48]. Similarly, the **InFlow** feasibility and usability study showed that active app usage significantly reduced ADHD symptoms [47]. Participants who completed **InFlow** were, on average, four years older than those who did not, with study participants aged 18–46.

Finally, in an acceptability study, researchers showed participants outlines of an **IBI** (without showing the full intervention), and participants reported that it would be more acceptable than a face-to-face version. This study also found that participants had no preference for tailored, individualised interventions, which was unexpected [55].

### Symptom monitoring

Symptom monitoring was the primary aim of four interventions. **StopWatch** and **Ecological Momentary Assessment** (**EMA**) aimed to improve ADHD symptoms using activity tracking and online surveys, respectively [50, 51]. Surman et al.’s SMS **Treatment Optimisation** intervention also used online surveys to monitor symptoms post-medication use, facilitating treatment optimisation for young people with their clinician [49]. The **FitBit Flex** app primarily aimed to help young people with ADHD reach physical activity goals and subsequently improve ADHD symptoms [52]. **StopWatch** and **FitBit Flex** were both delivered via smartwatches. While studies of symptom monitoring interventions have relatively high QuADS scores, they are all pilot or preliminary studies that did not use of control or comparator groups, reducing the level of certainty in the evidence.

All symptom monitoring studies collected preliminary intervention effects, feasibility and usability data. Study sample sizes ranged from 11 to 206 participants. Leikauf et al. used the parent-report ADHD-RS as an ADHD measure and qualitative interviews for feasibility data [51]. Schoenfelder et al. used the parent-report Vanderbilt ADHD Diagnostic Parent Rating Scale, adapted to collect data from adolescents, and also used the self-report 10-item Positive and Negative Affect Schedule for Children, daily step counts, app engagement data and qualitative feasibility questions [52]. Surman et al. used a subset of items from the ASRS, Weiss Functional Impairment Rating Scale and System Usability Scale [49]. Finally, Kennedy et al. collected data using non-validated measures of self-rated ADHD symptom severity (adapted from the Disruptive Behaviour Disorders scale and previous research) and monitored medication use daily [50].

Results indicated that symptom monitoring through **EMA** might improve ADHD symptoms, with lower self-reported symptom severity following the completion of 17-days of **EMA** [50]. Self-reported symptoms worsened throughout the day, especially during school days. Self-rated symptoms were lower following completion after a prompt versus completion following missing a prompt. The authors noted that this might indicate that **EMA** raises awareness of ADHD symptoms to the user and increases self-management efforts. However, this effect may also be because prompts are more likely to be missed when symptoms are worse, which is reflected in the subsequent measure. This study was also subject to a novelty effect, as symptom reductions were greatest in the first few days of the 17-day intervention.

Researchers explored whether the SMS **Treatment Optimisation** intervention could use self-report measures as a tool to monitor the effects of stimulant medication [49]. They found that the intervention was clinically sensitive, with good usability scores and a preference for the SMS intervention over a paper diary. These findings suggest that an SMS delivered monitoring intervention may be helpful in the monitoring and optimisation of medication regimes for people with ADHD and their clinicians.

**StopWatch** and the **FitBit Flex** app were both associated with improved ADHD symptoms, with decreases in parent-rated ADHD-RS scores and parent- and self-report VADPRS scores, respectively [51, 52]. A greater reduction in scores was observed for older participants (participant age range 8–17) [51], highlighting potential relevance for people at transition age [16–24, 28]. In addition to offering symptom monitoring, **FitBit Flex** also had components of social support allowing interaction with other participants via a private Facebook group [52]. Common improvements included better visual representations of data and user interfaces and more personalisation of thresholds, goals and targets [51, 52].

### Practical interventions

We only identified one practical intervention, explored in two studies [53, 54]. Biederman et al. tested the preliminary effectiveness and acceptability of **Medication Reminders**, an SMS intervention which reminded participants to take their stimulant medication, with findings indicating improved treatment adherence [53]. A second study, delivered in a primary care context, also found preliminary evidence of improved adherence and positive engagement with the intervention [54]. Both studies used TAU groups as a control and included large samples of participants, but with larger TAU than intervention groups (552 (Intervention = 92, TAU = 460); 448 (Intervention = 112, TAU = 336)). The intervention comprised text messages to participants with reminders to take their stimulants as prescribed; renew their prescriptions; and educational tips about treating ADHD and other self-management strategies. Researchers found that the intervention could be delivered in a primary care.

The studies assessed adherence to stimulant medication using a proxy measure of timely refill of prescriptions. Researchers regarded patients as engaging with treatment if refills of their prescriptions were documented in electronic medical records within a certain time frame. They excluded participants with serious medical or psychiatric conditions from both studies.

### Healthcare & self-management information

Two interventions provided healthcare & self-management information for young people with ADHD. One was a website (**IUEVO**), co-designed with adolescents [46], for educating youth about long-term conditions, including ADHD, and the other was a mobile app [56]. These studies provide the lowest level of evidence for all intervention categories, with **Luiu** et al.’s study scoring the lowest on the QuADS scale due to limited reporting as this was a peer-reviewed conference paper [56]. Wright et al.’s study also received a relatively low QuADS score [46]. In addition to the two interventions described here, all eight of the psychoeducation intervention also delivered healthcare & self-management information as part of psychoeducational modules [40–43, 47, 48, 55].

Both studies gathered qualitative data from a small number of participants (five in Wright et al.’s study, and six in Luiu et al.’s) who found the interventions acceptable and helpful. **IUEVO** was co-designed and included several iterative feedback phases, providing a useful framework for future development [46]. Suggestions included using specific formatting for people with ADHD, such as using large fonts and short bullet-point style sentences; the integration of multi-media (e.g., pictures and videos alongside text); and downloadable PDFs of content.

While these studies offer insights for future DHI development there is no evidence on whether standalone healthcare & self-management information reduces ADHD symptoms or improves self-management for young people aged 16–25.

## Discussion

### Summary

This review aimed to assess the quality of evidence from high income countries for DHIs that provide healthcare information, education and self-management resources/strategies to young people (aged 16–25) with ADHD. To our knowledge, it is the first systematic review focussed on peer-reviewed evidence of ADHD DHIs for this age group. 19 studies, exploring 15 interventions using heterogeneous methods, were included in this review. Although all studies involved participants with ADHD aged 16–25, none specifically targeted this population. Most participants were female, and approximately 75% were Caucasian.

Overall, findings indicate an emerging field of research, with evidence regarding the usability, feasibility and acceptability of different types of DHIs, but limited research of effectiveness. The strongest evidence came from five RCTs of psychoeducational DHIs, which found reductions in ADHD symptoms. But the identification of just five RCTs demonstrates a small evidence base, particularly for intervention categories other than psychoeducation. Pilot trials of DHIs focussed on symptom monitoring and practical tools provide preliminary evidence of effect. Quality assessment revealed that studies were of moderate to high quality.

This review also aimed to identify intervention components suitable for adaptation in UK-based primary care. While some promising components are indicated, none of the studies were conducted in the UK and few specifically examined the utility in a primary care setting, indicating the need for further research in this context.

### Main findings

#### Psychoeducation, symptom monitoring and medication reminders

Findings from the RCTs identified in this review suggest that psychoeducational DHIs are likely to reduce ADHD symptom severity, with clinically meaningful results [39–43]. This ties in with some research which demonstrates the effectiveness of some psychoeducational DHIs for the mental health of young people more broadly, although mixed evidence here suggests that the exact components and factors which illicit an effect require more investigation [67, 68]. There is also preliminary evidence which suggests that symptom monitoring using **EMA**, or wearable tracking devices, such as **StopWatch** or the **FitBit Flex** app, may help to reduce ADHD symptom severity [50–52]. For young people entering adulthood, gaining insight into their activity levels, and fluctuations in their ADHD symptoms may be an important aid for developing independence and learning to self-regulate and manage living with ADHD as a long-term condition [69]. Several ongoing trials are developing and trialling the promising field of symptom and activity tracking symptom as an aid to ADHD management [70, 71]. This review also found preliminary evidence indicating that SMS-based **Medication Reminders** may help with ADHD medication adherence [53, 54]. Improving adherence is a high priority, as ADHD medication is effective, reduces the risks of negative outcomes, and alongside non-pharmacological support can improve long-term management of ADHD [9, 10]. However, reducing systematic barriers to accessing medication should also remain a priority, as patient-facing DHIs will not overcome barriers such as medication shortages and long wait times [14]. It is also important to consider other unmet needs in mental health provision for young people with ADHD, beyond improving symptom severity and medication management. While this review focussed on health-related outcomes, and most DHIs included focussed only on symptom severity or medication adherence, there is growing recognition of the need to broaden the scope of interventions to address psychosocial aspects of care [72, 73]. Research highlights the importance of addressing unmet needs during transitional periods with treatment that goes beyond medication, such as interventions which focus on strengths and recovery [73, 74]. Future research should explore the integration of these approaches with DHIs identified in this review to enhance their relevance for young people and produce holistic interventions.

#### The need for self-management tools

Current UK evidence shows a higher-than-expected rate of discontinuation of medication in young people aged 16–25, therefore interventions to address this are a priority [75–77]. No evidence was found on the effectiveness of providing healthcare & self-management information alone on ADHD symptoms, in line with previous recommendations about the organisation of ADHD services [78]; although, every identified psychoeducational intervention provided this as part of their delivery [39–45, 47, 48, 55, 59]. As reflected in UK guidelines, research shows that healthcare information forms an important part of treatment and management of ADHD [12], however challenges with regulation of attention for young people with ADHD may mean that a theoretically informed approach, such as CBT is needed for this to have a positive impact [64]. This review did not find any evidence on whether provision of information alone (without psychoeducation) has a positive impact on ADHD symptoms in this age group. Assessing effectiveness of healthcare information alone is a priority as, if effective, provision of co-designed and accessible evidence-based healthcare information, like that provided on the **IUEVO** website [46], could represent a low-cost and scalable adjunct to usual care. Included studies used varied methods and incorporated a range of validated and un-validated measures, usually appropriate for their intended purpose. However, heterogeneity in study design and overlap between intervention categories mean there is not yet sufficient data to conduct a meta-analysis of RCT findings, or a robust evidence-base with which to determine the most effective intervention category or component.

#### Feasibility and acceptability of DHIs

Despite the identification of relatively few RCTs, a body of evidence was identified regarding the feasibility and acceptability of different types of DHIs for young people with ADHD, including usability and engagement data, which will be crucial for informing future development. Qualitative evidence on the overall acceptability and usability of DHIs was satisfactory, and in most cases when asked, participants stated a preference for DHIs over non-digital alternatives. Interventions developed using participatory methods (**MyADHD**) or co-design (**IUEVO**) provided the strongest evidence of usability, user-engagement, and acceptability [46, 59].

DHIs for symptom monitoring showed positive or moderate engagement, and positive acceptability, implying a potential for further development of these intervention types [49–52]. Research focussed on practical tools, SMS-based **Medication Reminders**, evidenced positive engagement from participants [53, 54]. Further research could explore other DHIs with practical tools to support self-management of ADHD, such as digital checklists to use in GP appointments as recommended by young people in MAP co-production work. Such intervention components are currently being developed by Sleath et al. [79, 80]. Findings from this systematic review and previous co-production work suggest that interactive elements of DHIs need to be carefully designed, working collaboratively with stakeholders, including young people with ADHD, to increase acceptability or engagement. It remains unclear whether all the suggested interactive elements should be a priority in the future development of DHIs.

#### Improving engagement and acceptability

One potential way of increasing engagement can be the use of SMS reminders. However, mixed evidence of SMS reminders on adherence to interventions was identified. No effect on adherence was found when used with the **MyADHD** app, but there was preliminary evidence of increased adherence with pharmacological treatment [44, 53, 54]. Despite limitations to the treatment adherence study, such as the use of prescription refills as proxy measures of adherence and a lack of long-term follow-up [53, 54], further exploration of the potential of co-designed reminders to improve engagement is indicated.

Another DHI component that might increase engagement and acceptability, is the use of conversational agents, or Chatbots, which use natural language processing to present psychoeducational content in optimal formats for patients [81]. Two included studies used Chatbots, with preliminary findings indicating reduced ADHD symptoms (though no better than conventional delivery) and variable evidence of acceptability [43, 48]. Mixed evidence in relation to interactive and text-messaging based adherence strategies suggests that they may not improve the overall benefits experienced from internet-based psychoeducation. This would benefit from further research,

A common limitation to DHIs was the unintuitive design of the user interface, conversational flow of Chatbot elements, or limited user engagement [43, 48, 51, 52]. Graphics and visuals used need to be up-to-date and appropriate for young people [51, 52]. Contrary to findings from Shelton et al.’s study, research also suggests that young people have a preference for personalised interventions and highlighted that emphasising strengths is a key priority of young people with ADHD [55, 73]. Therefore, designing DHIs with features which can be tailored to the individual, and which move away from the deficit model of care to increase engagement, and acceptability should be investigated. As DHIs progress at pace, including the development of components to facilitate engagement, such as conversational agents, wearable trackers, and SMS messaging, user-centred research will be important to optimise promising elements and address gaps in the evidence-base.

#### The importance of co-production and user-centric development

Improvements in usability scores of **MyADHD** following iterative refinement highlight the importance of optimising DHIs to the target population. Mixed evidence of feasibility for other interventions, this underlines the importance of including young people with ADHD in the development of DHIs and ties in with research showing that people with ADHD ask for, and prefer resources developed with people with lived experience of neurodiversity [82, 83]. Any intervention aiming to provide healthcare information and self-management resources for young people with ADHD needs first to engage their attention, be designed to be user-friendly for ‘ADHD brains’, and then find ways to promote continued engagement [82, 84]. Young people with ADHD can be particularly challenging to engage, due to organisational and attentional issues associated with having ADHD [5], so finding ways to increase engagement and usability (e.g. through co-production) is key for developing effective DHIs for this group. In addition, the evidence identified here fails to recruit diverse populations of young people with ADHD, with fewer than 20% of total participants belonging to minority ethnic groups. With systematic reviews highlighting the importance of developing culturally sensitive DHIs and interventions suitable for linguistically, socioeconomically and digitally marginalised young people [85–87], future research must focus on factors which may affect engagement with interventions for youth from minority backgrounds.

#### Implications for clinical practice

The results presented above suggest that DHIs which provide psychoeducation may be effective in reducing ADHD symptoms. However, included evidence does not specifically focus on the needs of people aged 16–25, or UK primary care settings, therefore findings should be interpreted with caution. This review did not find research evidence that DHIs providing self-management resources are more effective than pharmacological treatments. However, in line with UK guidelines, digital self-management resources may provide a useful adjunct to usual care for young people during the vulnerable life-stage of transition into adulthood [12], especially where access to ADHD healthcare in the UK remains limited [14, 20]. Research to support integration of DHIs to improve access to care for young people with long term conditions such as ADHD into general practice in the UK needs to be a priority [88]. Especially given NHS plans to redesign services and offer online solutions as a first port of call for support [28]. Barriers to implementing DHIs, such as clinician awareness and variable signposting of interventions will also need to be addressed as part of working towards digital inclusion [20].

#### Directions for future research and development

To progress this promising field, research needs to build on evidence identified in this review, particularly the development of the **MyADHD** app in Norway. Use of co-production methods with patients and clinicians from start to finish [89], clear methodological reporting, and using validated ADHD measures which triangulate self-report with clinical ratings where possible, is desirable. Future UK research needs to incorporate methodical intervention co-development, feasibility testing and evaluation, and trials of intervention components to aid engagement and effectiveness, with a clear focus on the unique needs and preferences of young people with ADHD, including those from minority backgrounds. Prioritising acceptability, usability and engagement will be critical to ensuring any DHIs designed for use in the UK are deliverable and can be implemented in primary care settings.

A rational and strategic approach to addressing gaps in the evidence base is needed. For example, prioritising evaluation of the impact of healthcare and self-management information provision alone on ADHD management, then incorporating this content into engaging and effective psychoeducational DHIs. Also collaborating with colleagues nationally, perhaps through the recently established NHS England Taskforce [90], to rapidly share learning. It is essential to streamline development and delivery of DHIs to enhance current delivery of healthcare for ADHD in the UK, which is in crisis [91]. As this field progresses, and the evidence builds, future systematic reviews will be able to conduct risk-of-bias assessments of trial evidence and provide meta-analyses of evidence of effectiveness in relation to ADHD symptom severity.

### Limitations

To our knowledge, this is the first systematic review of DHIs for young people aged 16–25 with ADHD, focussing on interventions which deliver information, tools and education but not neurocognitive training. This review identified and synthesised evidence from existing research using established and rigorous methods that are appropriate for exploring an emerging evidence base. However, there were limitations to methods used. In line with author’s expectations, and as indicated by our previous scoping work [24], included study designs were highly heterogenous, and included development, feasibility and pilot studies, with little effectiveness data. For this reason, the narrative synthesis approach used was appropriate to inform understanding of the available evidence [92]. Due to the heterogeneity of included studies and following consultation with the University of Exeter Evidence Synthesis team, appraisal of methodological and reporting quality was conducted using the QuADS tool, as this could be applied across all included studies and is increasingly being applied in mixed methods systematic reviews, because it demonstrates reliability, face and content validity across diverse types of evidence [36]. However, this tool does not include a risk of bias measure, meaning risk of bias was not assessed for the included trials.

Due to the limited research identified on DHIs of direct relevance for UK primary care contexts, our ability to address the second objective of this review was limited. However, evidence from this systematic review, such as the importance of co-development, is relevant for UK-based primary care settings and will form a robust foundation to inform future research and development.

Another limitation of this study is the broad inclusion criteria regarding participant age ranges. This decision was based on findings from a previous scoping review on the same topic [24], which found no studies of DHIs in which participant ages were entirely within the target age range of 16–25. The age range was selected due to its relevance for young people undergoing healthcare transitions who are commonly underserved in research, as highlighted by the scoping review. As such, studies were included if it could be assumed that at least one participant was within the target age range. This approach allowed for the inclusion of a broader selection of research with potential relevance for this age group, although it does introduce limitations regarding the specificity of findings for 16- to 25-year-olds. We acknowledge that having at least one participant within the target age range may not be sufficient to generalise findings directly to this population. This was a pragmatic decision, with an aim to include enough literature from this rapidly emerging field to draw preliminary insights into the existing research to guide the development of future DHIs for young people. This limitation demonstrates an important gap in the evidence base, underscoring the need for future research which explicitly focusses on young people.

Finally, to ensure the relevance of findings from this systematic review to UK healthcare settings, one of the inclusion criteria required that studies be from high-income countries. This criterion may have led to the exclusion of DHIs of interest from middle- or low-income countries that could be adapted to a UK healthcare setting. However, no studies were excluded on this basis (see Appendix 3 for reasons for exclusion).

## Conclusion

This systematic review provides initial data but also demonstrates a clear gap in the available evidence on DHIs suitable for young people aged 16–25 with ADHD. There is not yet enough evidence to assess the effectiveness of existing DHIs for use in the UK or to inform clinical practice recommendations. However, findings from this review can inform future research. They support development of CBT-based psychoeducational interventions, with content and user interfaces tailored for young people with ADHD. Interventions should include healthcare and self-management content and be designed to be accessible for people with variable attention spans. Digital components that might enhance engagement and strengthen impact, include symptom monitoring, using wearable devices and SMS messaging. The use of practical tools such as medication reminders, and interactive conversational assistants, should be investigated further. Co-producing future DHIs with stakeholders and adapting them to the needs of underserved populations (e.g. those from different ethnic or sociodemographic backgrounds) will be key to the future of inclusive digital healthcare for young people with ADHD. A more robust body of evidence is required to inform national adoption of DHIs to provide healthcare information and support self-management in young people with ADHD.

## Electronic supplementary material

Below is the link to the electronic supplementary material.

Supplementary Material 1

Supplementary Material 2

Supplementary Material 3

Supplementary Material 4

Supplementary Material 5

## Data Availability

No datasets were generated or analysed during the current study.
