# Correction: Digital health interventions with healthcare information and self-management resources for young people with ADHD: *a mixed-methods systematic review and narrative synthesis*

**DOI:** 10.1007/s00787-025-02727-4

**Published:** 2025-04-30

**Authors:** Rebecca Gudka, Elleie McGlynn, Katherine Lister, Naomi Shaw, Emma Pitchforth, Faraz Mughal, Blandine French, John Headly Ward, Tamsin Newlove-Delgado, Anna Price

**Affiliations:** 1 University of Exeter, Exeter, UK; 2 Keele University, Newcastle-Under-Lyme, UK; 3 University of Nottingham, Nottingham, UK; 4 University of Cambridge, Cambridge, UK

**Correction: European Child & Adolescent Psychiatry**

10.1007/s00787-025-02676-y

The Funding information section was missing from this article and should have read ‘This work was jointly funded through the NIHR SPCR project grant (FR6_640) held by AP, and through an NIHR Three Research Schools’ Mental Health Research Fellowship held by AP, located within Exeter SPCR (Grant Reference Number MHF008). JW is supported by the NIHR Oxford Health Biomedical Research Centre. TND was supported by an NIHR Advanced Fellowship during the preparation of this paper (NIHR300056). FM, doctoral fellow, is funded by NIHR (300957). The views expressed are those of the author(s) and not necessarily those of the NIHR, NHS or the Department of Health and Social Care.’

