# Supplementary material for: Digital health interventions with healthcare information and self-management resources for young people with ADHD: a mixed-methods systematic review and narrative synthesis
Source: Eur Child Adolesc Psychiatry. 2025 Mar 1;34(6):1817–35. doi: 10.1007/s00787-025-02676-y (PMC12198326; doi:10.1007/s00787-025-02676-y)
Supplement: Supplementary file 1 — Supplementary Material 1 [file 787_2025_2676_MOESM1_ESM.docx]

Digital health interventions including healthcare information and self-management resources for young people with ADHD: *A mixed-methods systematic review and narrative synthesis*

European Child & Adolescent Psychiatry

Rebecca Gudka*, Elleie McGlynn, Katherine Lister, Naomi Shaw, Emma Pitchforth, Faraz Mughal, Blandine French, John Headly Ward, Tamsin Newlove-Delgado, Anna Price

*[r.gudka@exeter.ac.uk](mailto:r.gudka@exeter.ac.uk) - University of Exeter (Faculty of Health and Life Sciences), Exeter, UK.

# Appendix 1. Search strategies

## MEDLINE

Ovid MEDLINE(R) ALL <1946 to August 03, 2023>

1 "Attention Deficit and Disruptive Behavior Disorders"/ or Attention Deficit Disorder with Hyperactivity/ 36759

2 ((attenti* or disrupt*) adj3 disorder*).ti,ab. 37952

3 (adhd or addh or ad hd or ad??hd).ti,ab. 31736

4 (attenti* adj3 deficit*).ti,ab. 39369

5 (((hyperkin* or hyper kin*) adj1 (syndrome* or disorder*)) or hkd).ti,ab. 997

6 1 or 2 or 3 or 4 or 5 [ADHD search terms derived from NICE guideline search strategies] 59116

7 Mobile Applications/ 11541

8 exp Internet/ 97707

9 exp Cell Phone/ 22449

10 exp Computers, Handheld/ 13084

11 Medical Informatics Applications/ 2551

12 Therapy, Computer-Assisted/ 6973

13 (app or apps).ti,ab. 43214

14 (online or web or internet or digital*).ti. 138494

15 ((online or web or internet or digital*) adj3 (based or application* or intervention* or program* or therap*)).ab. 79240

16 (phone* or telephone* or smartphone* or cellphone* or smartwatch*).ti. 27107

17 ((phone* or telephone* or smartphone* or cellphone* or smartwatch*) adj3 (based or application* or intervention* or program* or therap*)).ab. 16928

18 (mobile health or mhealth or m-health or ehealth or e-health or emental or e-mental).ti. 8497

19 ((mobile health or mhealth or m-health or ehealth or e-health or emental or e-mental) adj3 (based or application* or intervention* or program* or therap*)).ab. 5884

20 (mobile* adj3 (based or application* or intervention* or device* or technolog*)).ti,ab. 21814

21 or/7-20 [NICE health-application filter for MEDLINE] 343613

22 6 and 21 [ADHD terms combined with health apps filter] 906

23 computer-assisted instruction/ 12567

24 (computer* or software or technolog*).ti. 192027

25 ((digital* or online or internet* or web* or computer* or software or technolog*) adj3 (information or resource* or toolkit* or tool-kit* or aids or aids or guide* or interactiv* or advice or education* or psycho-education* or psychoeducation* or pathway*)).ab. 78299

26 ((digital* or online or internet* or web* or computer* or software or technolog*) adj3 (self-manag* or (self adj manag*) or self-monitor* or (self adj monitor*) or self-help* or (self adj help) or self-care or (self adj care) or adherence or reminder*)).ab. 3100

27 ((video* or multimedia* or multi-media* or audiovisual* or audio-visual* or youtube) adj3 (information or resource* or advice* or vignette* or story or stories or education* or psycho-education* or psychoeducation*)).ti,ab. 8033

28 (((text adj messag*) or SMS) and (self-manag* or (self adj manag*) or self-monitor* or (self adj monitor*) or self-help* or (self adj help) or self-care or (self adj care) or adherence or reminder* or medication*)).ti,ab. 3027

29 23 or 24 or 25 or 26 or 27 or 28 279297

30 Consumer Health Information/ 4305

31 ((health or patient) adj2 information).ti,ab. 56125

32 medication adherence/ 24258

33 self-management/ 5365

34 (self-manag* or (self adj manag*) or self-monitor* or (self adj monitor*) or self-care or (self adj care) or self-help or (self adj help)).ti,ab. 64581

35 ((medication or treatment) adj2 (adherence or reminder*)).ti,ab. 29962

36 30 or 31 or 32 or 33 or 34 or 35 163628

37 audiovisual aids/ or multimedia/ 9283

38 Social Media/ 15911

39 internet/ or internet-based intervention/ 82515

40 37 or 38 or 39 105138

41 36 and 40 9392

42 29 or 41 [digital information sources terms expanded] 283796

43 6 and 42 [ADHD terms combined with digital information resources terms] 455

44 22 or 43 [ADHD terms combined with both app terms or digital information resources] 1227

45 limit 44 to yr="2008 -Current" 1099

## Embase

Embase <1974 to 2023 August 03>

1 attention deficit hyperactivity disorder/ 9358

2 ((attenti* or disrupt*) adj3 disorder*).ti,ab. 49191

3 (adhd or addh or ad hd or ad??hd).ti,ab. 45757

4 (attenti* adj3 deficit*).ti,ab. 51296

5 (((hyperkin* or hyper kin*) adj1 (syndrome* or disorder*)) or hkd).ti,ab. 1433

6 1 or 2 or 3 or 4 or 5 75196

7 exp mobile application/ 25241

8 internet/ 123088

9 exp mobile phone/ 47149

10 text messaging/ 7619

11 personal digital assistant/ 1826

12 computer assisted therapy/ 4858

13 (app or apps).ti,ab. 58673

14 (online or web or internet or digital*).ti. 158383

15 ((online or web or internet or digital*) adj3 (based or application* or intervention* or program* or therap*)).ab. 106222

16 (phone* or telephone* or smartphone* or cellphone* or smartwatch*).ti. 32116

17 ((phone* or telephone* or smartphone* or cellphone* or smartwatch*) adj3 (based or application* or intervention* or program* or therap*)).ab. 22573

18 (mobile health or mhealth or m-health or ehealth or e-health or emental or e-mental).ti. 9336

19 ((mobile health or mhealth or m-health or ehealth or e-health or emental or e-mental) adj3 (based or application* or intervention* or program* or therap*)).ab. 6416

20 (mobile* adj3 (based or application* or intervention* or device* or technolog*)).ti,ab. 26783

21 or/7-20 439145

22 6 and 21 [ADHD with NICE apps terms] 1342

23 consumer health information/ 4267

24 ((health or patient) adj2 information).ti,ab. 77247

25 exp self care/ 101884

26 (self-manag* or (self adj manag*) or self-monitor* or (self adj monitor*) or self-help or (self adj help) or self-care or (self adj care)).ti,ab. 90181

27 ((medication or treatment) adj2 (adherence or reminder*)).ti,ab. 49934

28 or/23-27 259265

29 audiovisual aid/ 1018

30 multimedia/ 4950

31 social media/ 47790

32 internet/ or web-based intervention/ 125618

33 29 or 30 or 31 or 32 172548

34 28 and 33 15841

35 (computer* or software* or technolog*).ti. 230810

36 ((digital* or online or internet* or web* or computer* or software or technolog*) adj3 (information or resource* or toolkit* or tool-kit* or aids or aids or guide* or interactiv* or advice or education* or psycho-education* or psychoeducation* or pathway*)).ab. 93834

37 ((digital* or online or internet* or web* or computer* or software or technolog*) adj3 (self-manag* or (self adj manag*) or self-monitor* or (self adj monitor*) or self-help* or (self adj help) or self-care or (self adj care) or adherence or reminder*)).ab. 4044

38 ((video* or multimedia* or multi-media* or audiovisual* or audio-visual* or youtube) adj3 (information or resource* or advice or vignette* or story or stories or education* or psychoeducation* or psycho-education*)).ti,ab. 11580

39 (((text adj messag*) or SMS) and (self-manag* or (self adj manag*) or self-monitor* or (self adj monitor*) or self-help* or (self adj help) or self-care or (self adj care) or adherence or reminder* or medication*)).ti,ab. 4422

40 34 or 35 or 36 or 37 or 38 or 39 [DHI terms] 337379

41 6 and 40 624

42 22 or 41 1767

43 limit 42 to yr="2008 -Current" 1653

## PsycINFO

APA PsycInfo <1806 to July Week 4 2023>

1 exp Attention Deficit Disorder/ 31838

2 ((attenti* or disrupt*) adj3 disorder*).ti,ab. 35595

3 (adhd or addh or ad hd or ad??hd).ti,ab. 32769

4 (attenti* adj3 deficit*).ti,ab. 36830

5 (((hyperkin* or hyper kin*) adj1 (syndrome* or disorder*)) or hkd).ti,ab. 568

6 1 or 2 or 3 or 4 or 5 [ADHD search terms derived from NICE guideline search strategies] 50326

7 Mobile Applications/ 2456

8 exp Internet/ 31814

9 exp mobile devices/ 11127

10 computer assisted therapy/ 1231

11 (app or apps).ti,ab. 11035

12 (online or web or internet or digital*).ti. 59447

13 ((online or web or internet or digital*) adj3 (based or application* or intervention* or program* or therap*)).ab. 33054

14 (phone* or telephone* or smartphone* or cellphone* or smartwatch*).ti. 11453

15 ((phone* or telephone* or smartphone* or cellphone* or smartwatch*) adj3 (based or application* or intervention* or program* or therap*)).ab. 6016

16 (mobile health or mhealth or m-health or ehealth or e-health or emental or e-mental).ti. 1849

17 ((mobile health or mhealth or m-health or ehealth or e-health or emental or e-mental) adj3 (based or application* or intervention* or program* or therap*)).ab. 1513

18 (mobile* adj3 (based or application* or intervention* or device* or technolog*)).ti,ab. 8760

19 or/7-18 121588

20 6 and 19 [ADHD and adapted NICE app terms] 590

21 computer-assisted instruction/ 18499

22 digital interventions/ 1422

23 (computer* or software or technolog*).ti. 56144

24 ((digital* or online or internet* or web* or computer* or software or technolog*) adj3 (information or resource* or toolkit* or tool-kit* or aids or aids or guide* or interactiv* or advice or education* or psycho-education* or psychoeducation* or pathway*)).ab. 42380

25 ((digital* or online or internet* or web* or computer* or software or technolog*) adj3 (self-manag* or (self adj manag*) or self-monitor* or (self adj monitor*) or self-help* or (self adj help) or self-care or (self adj care) or adherence or reminder*)).ab. 1492

26 ((video* or multimedia* or multi-media* or audiovisual* or audio-visual* or youtube) adj3 (information or resource* or advice* or vignette* or story or stories or education* or psycho-education* or psychoeducation*)).ti,ab. 3985

27 (((text adj messag*) or SMS) and (self-manag* or (self adj manag*) or (self adj monitor*) or self-help* or (self adj help) or self-care or (self adj care) or adherence or reminder* or medication*)).ti,ab. 862

28 or/21-27 103818

29 exp Health Information/ 3516

30 ((health or patient) adj2 information).ti,ab. 12146

31 treatment compliance/ 17687

32 self-management/ or self-care/ or self-monitoring/ 14465

33 (self-manag* or (self adj manag*) or self-monitor* or (self adj monitor*) or self-care or (self adj care) or self-help or (self adj help)).ti,ab. 38366

34 ((medication or treatment) adj2 (adherence or reminder*)).ti,ab. 10655

35 or/29-34 75276

36 educational audiovisual aids/ or multimedia/ 3586

37 Social Media/ 15678

38 internet/ 31164

39 or/36-38 49192

40 35 and 39 2517

41 28 or 40 [Digital health terms] 104871

42 6 and 41 [ADHD and DHI terms] 456

43 20 or 42 [ADHD terms + apps or ADHD terms + DHI terms] 945

44 limit 43 to yr="2008 -Current" 801

## WoS

The Web of Science Core Collection databases covered by the search below include: Science Citation Index Expanded; Social Science Citation Index Expanded; Arts & Humanities Citation Index; Conference Proceedings Citation Index Science; Conference Proceedings Citation Index Social Science; Emerging Sources Citation Index.

# Web of Science Search Strategy (v0.1)

# Entitlements:

- WOS.SCI: 1900 to 2023

- WOS.AHCI: 1975 to 2023

- WOS.ESCI: 2015 to 2023

- WOS.ISTP: 1990 to 2023

- WOS.SSCI: 1900 to 2023

- WOS.ISSHP: 1990 to 2023

# Searches:

1: TS=((((hyperkin* or "hyper kin*") NEAR/1 (syndrome* or disorder*)) or hkd)) Results: 1938

2: TS=((adhd or addh or "ad hd")) Results: 44289

3: TS=((attenti* NEAR/2 deficit*)) Results: 54221

4: TS=(((attenti* or disrupt*) NEAR/2 disorder*)) Results: 51585

5: #1 OR #2 OR #3 OR #4 Results: 72412

6: TS=(((video* or multimedia* or multi-media* or audiovisual* or audio-visual* or youtube) NEAR/3 (information or resource* or advice* or vignette* or story or stories or education* or psycho-education* or psychoeducation*))) Results: 30164

7: AB=(((digital* or online or internet* or web* or computer* or software or technolog*) NEAR/3 (self-manag* or (self NEAR/1 manag*) or self-monitor* or (self NEAR/1 monitor*) or self-help* or adherence or reminder*))) Results: 4149

8: AB=(((digital* or online or internet* or web* or computer* or software or technolog*) NEAR/3 (information or resource* or toolkit* or tool-kit* or aids or aids or guide* or interactiv* or advice or education* or psycho-education* or psychoeducation* or pathway*))) Results: 379599

9: TI=((computer* or software or technolog*)) Results: 882393

10: #6 OR #7 OR #8 OR #9 Results: 1224139

11: #5 AND #10 Results: 754

12: TS=((mobile* near/3 (based or application* or intervention* or device* or technolog*))) Results: 149720

13: AB=((("mobile health" or mhealth or m-health or ehealth or e-health or emental or e-mental) near/3 (based or application* or intervention* or program* or therap*))) Results: 7354

14: TI=((mobile health or mhealth or m-health or ehealth or e-health or emental or e-mental)) Results: 14996

15: AB=(((phone* or telephone* or smartphone* or cellphone* or smartwatch*) near/3 (based or application* or intervention* or program* or therap*))) Results: 34033

16: TI=((phone* or telephone* or smartphone* or cellphone* or smartwatch*)) Results: 71669

17: AB=(((online or web or internet or digital*) NEAR/3 (based or application* or intervention* or program* or therap*))) Results: 246422

18: TI=((online or web or internet or digital*)) Results: 614814

19: TS=((app or apps)) Results: 86759

20: #12 OR #13 OR #14 OR #15 OR #16 OR #17 OR #18 OR #19 Results: 1044514

21: #20 AND #5 Results: 1107

22: #11 OR #21 Results: 1731

23: #11 OR #21 and 2023 or 2022 or 2021 or 2020 or 2019 or 2018 or 2017 or 2015 or 2014 or 2016 or 2013 or 2012 or 2011 or 2010 or 2009 or 2008 (Publication Years) Results: 1605

## Scopus – limited to 2008-2023

( ( ( TITLE ( ( computer*  OR  software  OR  technolog* ) ) )  OR  ( ABS ( ( ( digital*  OR  online  OR  internet*  OR  web*  OR  computer*  OR  software  OR  technolog* )  W/3  ( information  OR  resource*  OR  toolkit*  OR  tool-kit*  OR  aids  OR  aids  OR  guide*  OR  interactiv*  OR  advice  OR  education*  OR  psycho-education*  OR  psychoeducation*  OR  pathway* ) ) ) )  OR  ( ABS ( ( ( digital*  OR  online  OR  internet*  OR  web*  OR  computer*  OR  software  OR  technolog* )  W/3  ( self-manag*  OR  ( self  W/1  manag* )  OR  self-monitor*  OR  ( self  W/1  monitor* )  OR  self-help*  OR  ( self  W/1  help )  OR  self-care  OR  ( self  W/1  care )  OR  adherence  OR  reminder* ) ) ) )  OR  ( ( ABS ( ( ( video*  OR  multimedia*  OR  multi-media*  OR  audiovisual*  OR  audio-visual*  OR  youtube )  W/3  ( information  OR  resource*  OR  advice*  OR  vignette*  OR  story  OR  stories  OR  education*  OR  psycho-education*  OR  psychoeducation* ) ) )  OR  TITLE ( ( ( video*  OR  multimedia*  OR  multi-media*  OR  audiovisual*  OR  audio-visual*  OR  youtube )  W/3  ( information  OR  resource*  OR  advice*  OR  vignette*  OR  story  OR  stories  OR  education*  OR  psycho-education*  OR  psychoeducation* ) ) ) ) )  OR  ( ( ABS ( ( ( ( text  W/1  messag* )  OR  sms )  AND  ( self-manag*  OR  ( self  W/1  manag* )  OR  adherence  OR  reminder*  OR  medication* ) ) )  OR  TITLE ( ( ( ( text  W/1  messag* )  OR  sms )  AND  ( self-manag*  OR  ( self  W/1  manag* )  OR  adherence  OR  reminder*  OR  medication* ) ) ) ) ) )  OR  ( ( ( TITLE ( app  OR  apps )  OR  ABS ( app  OR  apps ) ) )  OR  ( TITLE ( ( online  OR  web  OR  internet  OR  digital* ) ) )  OR  ( ABS ( ( ( online  OR  web  OR  internet  OR  digital* )  W/3  ( based  OR  application*  OR  intervention*  OR  program*  OR  therap* ) ) ) )  OR  ( TITLE ( ( phone*  OR  telephone*  OR  smartphone*  OR  cellphone*  OR  smartwatch* ) ) )  OR  ( TITLE ( ( mobile  AND health  OR  mhealth  OR  m-health  OR  ehealth  OR  e-health  OR  emental  OR  e-mental ) ) ) ) )  AND  ( ( ( TITLE ( ( ( attenti*  OR  disrupt* )  W/3  disorder* ) )  OR  ABS ( ( ( attenti*  OR  disrupt* )  W/3  disorder* ) ) ) )  OR  ( ( TITLE ( ( attenti*  W/3  deficit* ) )  OR  ABS ( ( attenti*  W/3  deficit* ) ) ) )  OR  ( ( TITLE ( ( ( ( hyperkin* )  W/1  ( syndrome*  OR  disorder* ) )  OR  hkd ) )  OR  ABS ( ( ( ( hyperkin* )  W/1  ( syndrome*  OR  disorder* ) )  OR  hkd ) ) ) )  OR  ( ( TITLE ( ( adhd  OR  addh  OR  ad  AND hd ) )  OR  ABS ( ( adhd  OR  addh  OR  ad  AND hd ) ) ) ) ) 1368 records.

Limited to 2008-2023 (1231 records)

## ProQuest Dissertations & Theses Global

### ADHD and apps search

((title((app OR apps)) OR abstract((app OR apps))) OR title((online OR web OR internet OR digital*)) OR abstract(((online OR web OR internet OR digital*) NEAR/3 (based OR application* OR intervention* OR program* OR therap*))) OR title((phone* OR telephone* OR smartphone* OR cellphone* OR smartwatch*)) OR abstract(((phone* OR telephone* OR smartphone* OR cellphone* OR smartwatch*) NEAR/3 (based OR application* OR intervention* OR program* OR therap*))) OR title((mobile health OR mhealth OR m-health OR ehealth OR e-health OR emental OR e-mental)) OR abstract((("mobile health" OR mhealth OR m-health OR ehealth OR e-health OR emental OR e-mental) NEAR/3 (based OR application* OR intervention* OR program* OR therap*))) OR (abstract((mobile* NEAR/3 (based OR application* OR intervention* OR device* OR technolog*))) OR title((mobile* NEAR/3 (based OR application* OR intervention* OR device* OR technolog*))))) AND ((title(((attenti* OR disrupt*) NEAR/3 disorder*)) OR abstract(((attenti* OR disrupt*) NEAR/3 disorder*))) OR (title((adhd OR addh OR "ad hd")) OR abstract((adhd OR addh OR "ad hd"))) OR (title((attenti* NEAR/3 deficit*)) OR abstract((attenti* NEAR/3 deficit*))) OR (title(((hyperkin* NEAR/1 (syndrome* OR disorder*)) OR HKD)) OR abstract(((hyperkin* NEAR/1 (syndrome* OR disorder*)) OR HKD))))

Limited to 2008 – 2023

### ADHD and DHI search

((title(((attenti* OR disrupt*) NEAR/3 disorder*)) OR abstract(((attenti* OR disrupt*) NEAR/3 disorder*))) OR (title((adhd OR addh OR "ad hd")) OR abstract((adhd OR addh OR "ad hd"))) OR (title((attenti* NEAR/3 deficit*)) OR abstract((attenti* NEAR/3 deficit*))) OR (title(((hyperkin* NEAR/1 (syndrome* OR disorder*)) OR HKD)) OR abstract(((hyperkin* NEAR/1 (syndrome* OR disorder*)) OR HKD)))) AND (title((computer* OR software OR technolog*)) OR abstract(((digital* OR online OR internet* OR web* OR computer* OR software OR technolog*) NEAR/3 (information OR resource* OR toolkit* OR tool-kit* OR aids OR aids OR guide* OR interactiv* OR advice OR education* OR psycho-education* OR psychoeducation* OR pathway*))) OR abstract(((digital* OR online OR internet* OR web* OR computer* OR software OR technolog*) NEAR/3 (self-manag* OR (self NEAR/1 manag*) OR self-monitor* OR (self NEAR/1 monitor*) OR self-help* OR adherence OR reminder*))) OR (abstract(((video* OR multimedia* OR multi-media* OR audiovisual* OR audio-visual* OR youtube) NEAR/3 (information OR resource* OR advice* OR vignette* OR story OR stories OR education* OR psycho-education* OR psychoeducation*))) OR title(((video* OR multimedia* OR multi-media* OR audiovisual* OR audio-visual* OR youtube) NEAR/3 (information OR resource* OR advice* OR vignette* OR story OR stories OR education* OR psycho-education* OR psychoeducation*)))) OR (abstract((((text NEAR/1 messag*) OR SMS) AND (self-manag* OR (self NEAR/1 manag*) OR adherence OR reminder* OR medication*))) OR title((((text NEAR/1 messag*) OR SMS) AND (self-manag* OR (self NEAR/1 manag*) OR adherence OR reminder* OR medication*)))))

Limited to 2008-2023

## ACM Digital Library

### Search 1:

11 Results for: [[Title: adhd] OR [Title: addh] OR [Title: "attention deficit"] OR [Title: hyperkin*]] AND [[Title: app] OR [Title: apps] OR [Title: online] OR [Title: web] OR [Title: internet] OR [Title: digital*]] AND [E-Publication Date: (01/01/2008 TO 30/09/2023)]

### Search 2:

5 Results for: [[Title: adhd] OR [Title: addh] OR [Title: "attention deficit"] OR [Title: hyperkin*]] AND [[Title: phone*] OR [Title: telephone*] OR [Title: smartphone*] OR [Title: cellphone*] OR [Title: smartwatch*]] AND [E-Publication Date: (01/01/2008 TO 30/09/2023)]

### Search 3:

4 Results for: [[Title: adhd] OR [Title: addh] OR [Title: "attention deficit"] OR [Title: hyperkin*]] AND [[Title: "mobile health"] OR [Title: mhealth] OR [Title: m-health] OR [Title: ehealth] OR [Title: e-health] OR [Title: emental] OR [Title: e-mental]] AND [E-Publication Date: (01/01/2008 TO 30/09/2023)]

### Search 4:

1 Results for: [[Title: adhd] OR [Title: addh] OR [Title: "attention deficit"] OR [Title: hyperkin*]] AND [Title: mobile] AND [[Title: based] OR [Title: application*] OR [Title: intervention*] OR [Title: device*] OR [Title: technolog*]] AND [E-Publication Date: (01/01/2008 TO 30/09/2023)]

### Search 5:

22 Results for: [[Title: adhd] OR [Title: addh] OR [Title: "attention deficit"] OR [Title: hyperkin*]] AND [[Title: computer*] OR [Title: software] OR [Title: technolog*]] AND [E-Publication Date: (01/01/2008 TO 30/09/2023)]

### Search 6:

7 Results for: [[Title: adhd] OR [Title: addh] OR [Title: "attention deficit"] OR [Title: hyperkin*]] AND [[Title: video*] OR [Title: multimedia*] OR [Title: audiovisual*] OR [Title: youtube]] AND [E-Publication Date: (01/01/2008 TO 30/09/2023)]

## IEEE Xplore

### Search 1: (6 records)

("Document Title":adhd OR "Document Title":ADDH OR "Document Title":"attention deficit" OR "Document Title":hyperkin*) AND ("Document Title":app OR "Document Title":apps OR "Document Title":online OR "Document Title":web OR "Document Title":internet OR "Document Title":digital*)

### Search 2 (1 record)

("Document Title":adhd OR "Document Title":ADDH OR "Document Title":"attention deficit" OR "Document Title":hyperkin*) AND ("Document Title":phone* OR "Document Title":telephone* OR "Document Title":smartphone* OR "Document Title":cellphone* OR "Document Title":smartwatch*)

### Search 3: (1 record)

("Document Title":ADHD OR "Document Title":ADDH OR "Document Title":"attention deficit" OR "Document Title":hyperkin*) AND ("All Metadata":"mobile health" OR "All Metadata":mhealth OR "All Metadata":m-health OR "All Metadata":ehealth OR "All Metadata":e-health OR "All Metadata":emental OR "All Metadata":e-mental)

### Search 4: (5 records)

("Document Title":ADDH OR "Document Title":ADHD OR "Document Title":"attention deficit" OR "Document Title":hyperkin*) AND ("Document Title":mobile*)

### Search 5: (15 records)

("Document Title":ADDH OR "Document Title":ADHD OR "Document Title":"attention deficit" OR "Document Title":hyperkin*) AND ("Document Title":computer* OR "Document Title":software* OR "Document Title":technolog*)

### Search 6: (6 records)

("Document Title":ADDH OR "Document Title":ADHD OR "Document Title":"attention deficit" OR "Document Title":hyperkin*) AND ("Document Title":video* OR "Document Title":multimedia* OR "Document Title":audiovisual* OR "Document Title":youtube)

#

# Update searches: 15 December 2023

## Ovid MEDLINE

Ovid MEDLINE(R) ALL <1946 to December 07, 2023>

1 "Attention Deficit and Disruptive Behavior Disorders"/ or Attention Deficit Disorder with Hyperactivity/ 37278

2 ((attenti* or disrupt*) adj3 disorder*).ti,ab. 38898

3 (adhd or addh or ad hd or ad??hd).ti,ab. 32568

4 (attenti* adj3 deficit*).ti,ab. 40296

5 (((hyperkin* or hyper kin*) adj1 (syndrome* or disorder*)) or hkd).ti,ab. 1004

6 1 or 2 or 3 or 4 or 5 [ADHD search terms derived from NICE guideline search strategies] 60363

7 Mobile Applications/ 11954

8 exp Internet/ 98974

9 exp Cell Phone/ 22968

10 exp Computers, Handheld/ 13447

11 Medical Informatics Applications/ 2552

12 Therapy, Computer-Assisted/ 6979

13 (app or apps).ti,ab. 45084

14 (online or web or internet or digital*).ti. 143737

15 ((online or web or internet or digital*) adj3 (based or application* or intervention* or program* or therap*)).ab. 82607

16 (phone* or telephone* or smartphone* or cellphone* or smartwatch*).ti. 27922

17 ((phone* or telephone* or smartphone* or cellphone* or smartwatch*) adj3 (based or application* or intervention* or program* or therap*)).ab. 17628

18 (mobile health or mhealth or m-health or ehealth or e-health or emental or e-mental).ti. 8891

19 ((mobile health or mhealth or m-health or ehealth or e-health or emental or e-mental) adj3 (based or application* or intervention* or program* or therap*)).ab. 6223

20 (mobile* adj3 (based or application* or intervention* or device* or technolog*)).ti,ab. 22839

21 or/7-20 [NICE health-application filter for MEDLINE] 354901

22 6 and 21 [ADHD terms combined with health apps filter] 946

23 computer-assisted instruction/ 12612

24 (computer* or software or technolog*).ti. 196347

25 ((digital* or online or internet* or web* or computer* or software or technolog*) adj3 (information or resource* or toolkit* or tool-kit* or aids or aids or guide* or interactiv* or advice or education* or psycho-education* or psychoeducation* or pathway*)).ab. 81389

26 ((digital* or online or internet* or web* or computer* or software or technolog*) adj3 (self-manag* or (self adj manag*) or self-monitor* or (self adj monitor*) or self-help* or (self adj help) or self-care or (self adj care) or adherence or reminder*)).ab. 3224

27 ((video* or multimedia* or multi-media* or audiovisual* or audio-visual* or youtube) adj3 (information or resource* or advice* or vignette* or story or stories or education* or psycho-education* or psychoeducation*)).ti,ab. 8403

28 (((text adj messag*) or SMS) and (self-manag* or (self adj manag*) or self-monitor* or (self adj monitor*) or self-help* or (self adj help) or self-care or (self adj care) or adherence or reminder* or medication*)).ti,ab. 3163

29 23 or 24 or 25 or 26 or 27 or 28 287025

30 Consumer Health Information/ 4323

31 ((health or patient) adj2 information).ti,ab. 57916

32 medication adherence/ 24544

33 self-management/ 5588

34 (self-manag* or (self adj manag*) or self-monitor* or (self adj monitor*) or self-care or (self adj care) or self-help or (self adj help)).ti,ab. 66669

35 ((medication or treatment) adj2 (adherence or reminder*)).ti,ab. 30984

36 30 or 31 or 32 or 33 or 34 or 35 168473

37 audiovisual aids/ or multimedia/ 9308

38 Social Media/ 16541

39 internet/ or internet-based intervention/ 83116

40 37 or 38 or 39 106385

41 36 and 40 9529

42 29 or 41 [digital information sources terms expanded] 291587

43 6 and 42 [ADHD terms combined with digital information resources terms] 473

44 22 or 43 [ADHD terms combined with both app terms or digital information resources] 1281

45 limit 44 to yr="2008-Current" 1153

## Embase

Embase <1974 to 2023 December 13>

1 attention deficit hyperactivity disorder/ 11775

2 ((attenti* or disrupt*) adj3 disorder*).ti,ab. 50501

3 (adhd or addh or ad hd or ad??hd).ti,ab. 47225

4 (attenti* adj3 deficit*).ti,ab. 52590

5 (((hyperkin* or hyper kin*) adj1 (syndrome* or disorder*)) or hkd).ti,ab. 1453

6 1 or 2 or 3 or 4 or 5 77964

7 exp mobile application/ 26662

8 internet/ 124825

9 exp mobile phone/ 49261

10 text messaging/ 7912

11 personal digital assistant/ 1842

12 computer assisted therapy/ 4864

13 (app or apps).ti,ab. 61201

14 (online or web or internet or digital*).ti. 164013

15 ((online or web or internet or digital*) adj3 (based or application* or intervention* or program* or therap*)).ab. 110119

16 (phone* or telephone* or smartphone* or cellphone* or smartwatch*).ti. 32983

17 ((phone* or telephone* or smartphone* or cellphone* or smartwatch*) adj3 (based or application* or intervention* or program* or therap*)).ab. 23407

18 (mobile health or mhealth or m-health or ehealth or e-health or emental or e-mental).ti. 9715

19 ((mobile health or mhealth or m-health or ehealth or e-health or emental or e-mental) adj3 (based or application* or intervention* or program* or therap*)).ab. 6744

20 (mobile* adj3 (based or application* or intervention* or device* or technolog*)).ti,ab. 27921

21 or/7-20 453264

22 6 and 21 [ADHD with NICE apps terms] 1450

23 consumer health information/ 4303

24 ((health or patient) adj2 information).ti,ab. 79398

25 exp self care/ 104639

26 (self-manag* or (self adj manag*) or self-monitor* or (self adj monitor*) or self-help or (self adj help) or self-care or (self adj care)).ti,ab. 93000

27 ((medication or treatment) adj2 (adherence or reminder*)).ti,ab. 51655

28 or/23-27 266603

29 audiovisual aid/ 1036

30 multimedia/ 5029

31 social media/ 51251

32 internet/ or web-based intervention/ 127604

33 29 or 30 or 31 or 32 177808

34 28 and 33 16320

35 (computer* or software* or technolog*).ti. 235490

36 ((digital* or online or internet* or web* or computer* or software or technolog*) adj3 (information or resource* or toolkit* or tool-kit* or aids or aids or guide* or interactiv* or advice or education* or psycho-education* or psychoeducation* or pathway*)).ab. 96833

37 ((digital* or online or internet* or web* or computer* or software or technolog*) adj3 (self-manag* or (self adj manag*) or self-monitor* or (self adj monitor*) or self-help* or (self adj help) or self-care or (self adj care) or adherence or reminder*)).ab. 4213

38 ((video* or multimedia* or multi-media* or audiovisual* or audio-visual* or youtube) adj3 (information or resource* or advice or vignette* or story or stories or education* or psychoeducation* or psycho-education*)).ti,ab. 12081

39 (((text adj messag*) or SMS) and (self-manag* or (self adj manag*) or self-monitor* or (self adj monitor*) or self-help* or (self adj help) or self-care or (self adj care) or adherence or reminder* or medication*)).ti,ab. 4605

40 34 or 35 or 36 or 37 or 38 or 39 [DHI terms] 345800

41 6 and 40 656

42 22 or 41 1895

43 limit 42 to yr="2008-Current" 1781

## PsycINFO

APA PsycInfo <1806 to December Week 1 2023>

1 exp Attention Deficit Disorder/ 32415

2 ((attenti* or disrupt*) adj3 disorder*).ti,ab. 36168

3 (adhd or addh or ad hd or ad??hd).ti,ab. 33352

4 (attenti* adj3 deficit*).ti,ab. 37412

5 (((hyperkin* or hyper kin*) adj1 (syndrome* or disorder*)) or hkd).ti,ab. 571

6 1 or 2 or 3 or 4 or 5 [ADHD search terms derived from NICE guideline search strategies] 51125

7 Mobile Applications/ 2748

8 exp Internet/ 33896

9 exp mobile devices/ 11833

10 computer assisted therapy/ 1250

11 (app or apps).ti,ab. 11661

12 (online or web or internet or digital*).ti. 61866

13 ((online or web or internet or digital*) adj3 (based or application* or intervention* or program* or therap*)).ab. 34305

14 (phone* or telephone* or smartphone* or cellphone* or smartwatch*).ti. 11782

15 ((phone* or telephone* or smartphone* or cellphone* or smartwatch*) adj3 (based or application* or intervention* or program* or therap*)).ab. 6237

16 (mobile health or mhealth or m-health or ehealth or e-health or emental or e-mental).ti. 1960

17 ((mobile health or mhealth or m-health or ehealth or e-health or emental or e-mental) adj3 (based or application* or intervention* or program* or therap*)).ab. 1611

18 (mobile* adj3 (based or application* or intervention* or device* or technolog*)).ti,ab. 9117

19 or/7-18 126470

20 6 and 19 [ADHD and adapted NICE app terms] 624

21 computer-assisted instruction/ 18732

22 digital interventions/ 1682

23 (computer* or software or technolog*).ti. 57083

24 ((digital* or online or internet* or web* or computer* or software or technolog*) adj3 (information or resource* or toolkit* or tool-kit* or aids or aids or guide* or interactiv* or advice or education* or psycho-education* or psychoeducation* or pathway*)).ab. 43526

25 ((digital* or online or internet* or web* or computer* or software or technolog*) adj3 (self-manag* or (self adj manag*) or self-monitor* or (self adj monitor*) or self-help* or (self adj help) or self-care or (self adj care) or adherence or reminder*)).ab. 1555

26 ((video* or multimedia* or multi-media* or audiovisual* or audio-visual* or youtube) adj3 (information or resource* or advice* or vignette* or story or stories or education* or psycho-education* or psychoeducation*)).ti,ab. 4123

27 (((text adj messag*) or SMS) and (self-manag* or (self adj manag*) or (self adj monitor*) or self-help* or (self adj help) or self-care or (self adj care) or adherence or reminder* or medication*)).ti,ab. 896

28 or/21-27 106187

29 exp Health Information/ 3739

30 ((health or patient) adj2 information).ti,ab. 12451

31 treatment compliance/ 17974

32 self-management/ or self-care/ or self-monitoring/ 14824

33 (self-manag* or (self adj manag*) or self-monitor* or (self adj monitor*) or self-care or (self adj care) or self-help or (self adj help)).ti,ab. 39230

34 ((medication or treatment) adj2 (adherence or reminder*)).ti,ab. 10890

35 or/29-34 76938

36 educational audiovisual aids/ or multimedia/ 3659

37 Social Media/ 16905

38 internet/ 31379

39 or/36-38 50676

40 35 and 39 2578

41 28 or 40 [Digital health terms] 107279

42 6 and 41 [ADHD and DHI terms] 467

43 20 or 42 [ADHD terms + apps or ADHD terms + DHI terms] 986

44 limit 43 to yr="2008-Current" 842

## Web of Science

# Web of Science Search Strategy (v0.1) 15/12/2023

#1 TS=((((hyperkin* or "hyper kin*") NEAR/1 (syndrome* or disorder*)) or hkd)) Results: 1965

#2 Search: TS=((adhd or addh or "ad hd")) Date Run: Fri Dec 15 2023 16:26:35 GMT+0000 (Greenwich Mean Time) Results: 45231

#3 Search: TS=((attenti* NEAR/2 deficit*)) Date Run: Fri Dec 15 2023 16:26:35 GMT+0000 (Greenwich Mean Time) Results: 55427

#4 Search: TS=(((attenti* or disrupt*) NEAR/2 disorder*)) Date Run: Fri Dec 15 2023 16:26:36 GMT+0000 (Greenwich Mean Time) Results: 52805

#5 Search: #1 OR #2 OR #3 OR #4 Date Run: Fri Dec 15 2023 16:26:36 GMT+0000 (Greenwich Mean Time) Results: 74105

#6 Search: TS=(((video* or multimedia* or multi-media* or audiovisual* or audio-visual* or youtube) NEAR/3 (information or resource* or advice* or vignette* or story or stories or education* or psycho-education* or psychoeducation*))) Date Run: Fri Dec 15 2023 16:26:37 GMT+0000 (Greenwich Mean Time) Results: 31099

#7 Search: AB=(((digital* or online or internet* or web* or computer* or software or technolog*) NEAR/3 (self-manag* or (self NEAR/1 manag*) or self-monitor* or (self NEAR/1 monitor*) or self-help* or adherence or reminder*))) Date Run: Fri Dec 15 2023 16:26:37 GMT+0000 (Greenwich Mean Time) Results: 4313

#8 Search: AB=(((digital* or online or internet* or web* or computer* or software or technolog*) NEAR/3 (information or resource* or toolkit* or tool-kit* or aids or aids or guide* or interactiv* or advice or education* or psycho-education* or psychoeducation* or pathway*))) Date Run: Fri Dec 15 2023 16:26:38 GMT+0000 (Greenwich Mean Time) Results: 389661

#9 Search: TI=((computer* or software or technolog*)) Date Run: Fri Dec 15 2023 16:26:38 GMT+0000 (Greenwich Mean Time) Results: 897054

#10 Search: #6 OR #7 OR #8 OR #9 Date Run: Fri Dec 15 2023 16:26:39 GMT+0000 (Greenwich Mean Time) Results: 1248351

#11 Search: #5 AND #10 Date Run: Fri Dec 15 2023 16:26:39 GMT+0000 (Greenwich Mean Time) Results: 782

#12 Search: TS=((mobile* near/3 (based or application* or intervention* or device* or technolog*))) Date Run: Fri Dec 15 2023 16:26:40 GMT+0000 (Greenwich Mean Time) Results: 153325

#13 Search: AB=((("mobile health" or mhealth or m-health or ehealth or e-health or emental or e-mental) near/3 (based or application* or intervention* or program* or therap*))) Date Run: Fri Dec 15 2023 16:26:41 GMT+0000 (Greenwich Mean Time) Results: 7732

#14 Search: TI=((mobile health or mhealth or m-health or ehealth or e-health or emental or e-mental)) Date Run: Fri Dec 15 2023 16:26:41 GMT+0000 (Greenwich Mean Time) Results: 15683

#15 Search: AB=(((phone* or telephone* or smartphone* or cellphone* or smartwatch*) near/3 (based or application* or intervention* or program* or therap*))) Date Run: Fri Dec 15 2023 16:26:41 GMT+0000 (Greenwich Mean Time) Results: 35259

#16 Search: TI=((phone* or telephone* or smartphone* or cellphone* or smartwatch*)) Date Run: Fri Dec 15 2023 16:26:42 GMT+0000 (Greenwich Mean Time) Results: 73334

#17 Search: AB=(((online or web or internet or digital*) NEAR/3 (based or application* or intervention* or program* or therap*))) Date Run: Fri Dec 15 2023 16:26:42 GMT+0000 (Greenwich Mean Time) Results: 253965

#18 Search: TI=((online or web or internet or digital*)) Date Run: Fri Dec 15 2023 16:26:43 GMT+0000 (Greenwich Mean Time) Results: 632695

#19 Search: TS=((app or apps)) Date Run: Fri Dec 15 2023 16:26:43 GMT+0000 (Greenwich Mean Time) Results: 89415

#20 Search: #12 OR #13 OR #14 OR #15 OR #16 OR #17 OR #18 OR #19 Date Run: Fri Dec 15 2023 16:26:44 GMT+0000 (Greenwich Mean Time) Results: 1073596

#21 Search: #20 AND #5 Date Run: Fri Dec 15 2023 16:26:44 GMT+0000 (Greenwich Mean Time) Results: 1183

#22 Search: #11 OR #21 Date Run: Fri Dec 15 2023 16:26:45 GMT+0000 (Greenwich Mean Time) Results: 1827

#23 Search: #11 OR #21 and 2023 or 2022 or 2021 or 2020 or 2019 or 2018 or 2017 or 2016 or 2015 or 2014 or 2013 or 2012 or 2011 or 2010 or 2009 or 2008 (Publication Years) Date Run: Fri Dec 15 2023 16:27:09 GMT+0000 (Greenwich Mean Time) Results: 1702

# Database: Web of Science Core Collection

# Entitlements:

- WOS.SCI: 1900 to 2023

- WOS.AHCI: 1975 to 2023

- WOS.ESCI: 2015 to 2023

- WOS.ISTP: 1990 to 2023

- WOS.SSCI: 1900 to 2023

- WOS.ISSHP: 1990 to 2023

## Scopus

15/12/2023

( ( ( TITLE ( ( computer* OR software OR technolog* ) ) ) OR ( ABS ( ( ( digital* OR online OR internet* OR web* OR computer* OR software OR technolog* ) W/3 ( information OR resource* OR toolkit* OR tool-kit* OR aids OR aids OR guide* OR interactiv* OR advice OR education* OR psycho-education* OR psychoeducation* OR pathway* ) ) ) ) OR ( ABS ( ( ( digital* OR online OR internet* OR web* OR computer* OR software OR technolog* ) W/3 ( self-manag* OR ( self W/1 manag* ) OR self-monitor* OR ( self W/1 monitor* ) OR self-help* OR ( self W/1 help ) OR self-care OR ( self W/1 care ) OR adherence OR reminder* ) ) ) ) OR ( ( ABS ( ( ( video* OR multimedia* OR multi-media* OR audiovisual* OR audio-visual* OR youtube ) W/3 ( information OR resource* OR advice* OR vignette* OR story OR stories OR education* OR psycho-education* OR psychoeducation* ) ) ) OR TITLE ( ( ( video* OR multimedia* OR multi-media* OR audiovisual* OR audio-visual* OR youtube ) W/3 ( information OR resource* OR advice* OR vignette* OR story OR stories OR education* OR psycho-education* OR psychoeducation* ) ) ) ) ) OR ( ( ABS ( ( ( ( text W/1 messag* ) OR sms ) AND ( self-manag* OR ( self W/1 manag* ) OR adherence OR reminder* OR medication* ) ) ) OR TITLE ( ( ( ( text W/1 messag* ) OR sms ) AND ( self-manag* OR ( self W/1 manag* ) OR adherence OR reminder* OR medication* ) ) ) ) ) ) OR ( ( ( TITLE ( app OR apps ) OR ABS ( app OR apps ) ) ) OR ( TITLE ( ( online OR web OR internet OR digital* ) ) ) OR ( ABS ( ( ( online OR web OR internet OR digital* ) W/3 ( based OR application* OR intervention* OR program* OR therap* ) ) ) ) OR ( TITLE ( ( phone* OR telephone* OR smartphone* OR cellphone* OR smartwatch* ) ) ) OR ( TITLE ( ( mobile AND health OR mhealth OR m-health OR ehealth OR e-health OR emental OR e-mental ) ) ) ) ) AND ( ( ( TITLE ( ( ( attenti* OR disrupt* ) W/3 disorder* ) ) OR ABS ( ( ( attenti* OR disrupt* ) W/3 disorder* ) ) ) ) OR ( ( TITLE ( ( attenti* W/3 deficit* ) ) OR ABS ( ( attenti* W/3 deficit* ) ) ) ) OR ( ( TITLE ( ( ( ( hyperkin* ) W/1 ( syndrome* OR disorder* ) ) OR hkd ) ) OR ABS ( ( ( ( hyperkin* ) W/1 ( syndrome* OR disorder* ) ) OR hkd ) ) ) ) OR ( ( TITLE ( ( adhd OR addh OR ad AND hd ) ) OR ABS ( ( adhd OR addh OR ad AND hd ) ) ) ) ) AND PUBYEAR > 2007 AND PUBYEAR < 2025

## ProQuest Dissertations & Theses

Search date 15/12/2023

### ADHD + DHI search

((title(((attenti* OR disrupt*) NEAR/3 disorder*)) OR abstract(((attenti* OR disrupt*) NEAR/3 disorder*))) OR (title((adhd OR addh OR "ad hd")) OR abstract((adhd OR addh OR "ad hd"))) OR (title((attenti* NEAR/3 deficit*)) OR abstract((attenti* NEAR/3 deficit*))) OR (title(((hyperkin* NEAR/1 (syndrome* OR disorder*)) OR HKD)) OR abstract(((hyperkin* NEAR/1 (syndrome* OR disorder*)) OR HKD)))) AND (title((computer* OR software OR technolog*)) OR abstract(((digital* OR online OR internet* OR web* OR computer* OR software OR technolog*) NEAR/3 (information OR resource* OR toolkit* OR tool-kit* OR aids OR aids OR guide* OR interactiv* OR advice OR education* OR psycho-education* OR psychoeducation* OR pathway*))) OR abstract(((digital* OR online OR internet* OR web* OR computer* OR software OR technolog*) NEAR/3 (self-manag* OR (self NEAR/1 manag*) OR self-monitor* OR (self NEAR/1 monitor*) OR self-help* OR adherence OR reminder*))) OR (abstract(((video* OR multimedia* OR multi-media* OR audiovisual* OR audio-visual* OR youtube) NEAR/3 (information OR resource* OR advice* OR vignette* OR story OR stories OR education* OR psycho-education* OR psychoeducation*))) OR title(((video* OR multimedia* OR multi-media* OR audiovisual* OR audio-visual* OR youtube) NEAR/3 (information OR resource* OR advice* OR vignette* OR story OR stories OR education* OR psycho-education* OR psychoeducation*)))) OR (abstract((((text NEAR/1 messag*) OR SMS) AND (self-manag* OR (self NEAR/1 manag*) OR adherence OR reminder* OR medication*))) OR title((((text NEAR/1 messag*) OR SMS) AND (self-manag* OR (self NEAR/1 manag*) OR adherence OR reminder* OR medication*)))))

### ADHD + apps

15/12/2023

((title((app OR apps)) OR abstract((app OR apps))) OR title((online OR web OR internet OR digital*)) OR abstract(((online OR web OR internet OR digital*) NEAR/3 (based OR application* OR intervention* OR program* OR therap*))) OR title((phone* OR telephone* OR smartphone* OR cellphone* OR smartwatch*)) OR abstract(((phone* OR telephone* OR smartphone* OR cellphone* OR smartwatch*) NEAR/3 (based OR application* OR intervention* OR program* OR therap*))) OR title((mobile health OR mhealth OR m-health OR ehealth OR e-health OR emental OR e-mental)) OR abstract((("mobile health" OR mhealth OR m-health OR ehealth OR e-health OR emental OR e-mental) NEAR/3 (based OR application* OR intervention* OR program* OR therap*))) OR (abstract((mobile* NEAR/3 (based OR application* OR intervention* OR device* OR technolog*))) OR title((mobile* NEAR/3 (based OR application* OR intervention* OR device* OR technolog*))))) AND ((title(((attenti* OR disrupt*) NEAR/3 disorder*)) OR abstract(((attenti* OR disrupt*) NEAR/3 disorder*))) OR (title((adhd OR addh OR "ad hd")) OR abstract((adhd OR addh OR "ad hd"))) OR (title((attenti* NEAR/3 deficit*)) OR abstract((attenti* NEAR/3 deficit*))) OR (title(((hyperkin* NEAR/1 (syndrome* OR disorder*)) OR HKD)) OR abstract(((hyperkin* NEAR/1 (syndrome* OR disorder*)) OR HKD)))) AND yr(2008-2023)

## ACM Digital Library

### Search 1: 43 results

[[Title: adhd] OR [Title: addh] OR [Title: "attention deficit"] OR [Title: hyperkin*]] AND [[Title: app] OR [Title: apps] OR [Title: online] OR [Title: web] OR [Title: internet] OR [Title: digital*]] AND [E-Publication Date: (01/01/2008 TO 31/12/2024)]

### Search 2: 8 results

 [[Title: adhd] OR [Title: addh] OR [Title: "attention deficit"] OR [Title: hyperkin*]] AND [[Title: phone*] OR [Title: telephone*] OR [Title: smartphone*] OR [Title: cellphone*] OR [Title: smartwatch*]] AND [E-Publication Date: (01/01/2008 TO 31/12/2024)]

### Search 3: 9 results

[[Title: adhd] OR [Title: addh] OR [Title: "attention deficit"] OR [Title: hyperkin*]] AND [[Title: "mobile health"] OR [Title: mhealth] OR [Title: m-health] OR [Title: ehealth] OR [Title: e-health] OR [Title: emental] OR [Title: e-mental]] AND [E-Publication Date: (01/01/2008 TO 31/12/2024)]

### Search 4: 3 results

[[Title: adhd] OR [Title: addh] OR [Title: "attention deficit"] OR [Title: hyperkin*]] AND [Title: mobile] AND [[Title: based] OR [Title: application*] OR [Title: intervention*] OR [Title: device*] OR [Title: technolog*]] AND [E-Publication Date: (01/01/2008 TO 31/12/2024)]

### Search 5: 47 results

[Title: adhd] OR [Title: addh] OR [Title: "attention deficit"] OR [Title: hyperkin*]] AND [[Title: computer*] OR [Title: software] OR [Title: technolog*]] AND [E-Publication Date: (01/01/2008 TO 31/12/2024)]

### Search 6: 19 results

 [[Title: adhd] OR [Title: addh] OR [Title: "attention deficit"] OR [Title: hyperkin*]] AND [[Title: video*] OR [Title: multimedia*] OR [Title: audiovisual*] OR [Title: youtube]] AND [E-Publication Date: (01/01/2008 TO 31/12/2024)]

## IEEE Xplore

15^th^ December 2023

### Search 1: 8 results

("Document Title":adhd OR "Document Title":addh OR "Document Title":"attention deficit" OR "Document Title":hyperkin*) AND ("Document Title":app OR "Document Title":apps OR "Document Title":online OR "Document Title":web OR "Document Title":internet OR "Document Title":digital)

### Search 2: 2 results

("Document Title":adhd OR "Document Title":ADDH OR "Document Title":"attention deficit" OR "Document Title":hyperkin*) AND ("Document Title":phone* OR "Document Title":telephone* OR "Document Title":smartphone* OR "Document Title":cellphone* OR "Document Title":smartwatch*)

### Search 3: 0 results

("Document Title":adhd OR "Document Title":Addh OR "Document Title":attention deficit or hyperkin*) AND ("All Metadata":mobile health or mhealth or m-health or ehealth or e-health or e-mental)

### Search 4: 6 results

("Document Title":addh OR "Document Title":adhd OR "Document Title":attention deficit OR "Document Title":hyperkin*) AND ("Document Title":mobile*)

### Search 5: 0 results

("Document Title":ADDH OR "Document Title":ADHD OR "Document Title":attention deficit OR "Document Title":hyperkin*) AND ("Document Title":computer* or software* or technolog*)

### Search 6: 0 results

("Document Title":adhd OR "Document Title":ADHD OR "Document Title":attention deficit OR "Document Title":hyperkin*) AND ("Document Title":video* or multimedia* or audiovisual* or youtube)
