# Supplementary material for: Digital health interventions with healthcare information and self-management resources for young people with ADHD: a mixed-methods systematic review and narrative synthesis
Source: Eur Child Adolesc Psychiatry. 2025 Mar 1;34(6):1817–35. doi: 10.1007/s00787-025-02676-y (PMC12198326; doi:10.1007/s00787-025-02676-y)
Supplement: Supplementary file 2 — Supplementary Material 2 [file 787_2025_2676_MOESM2_ESM.docx]

Digital health interventions including healthcare information and self-management resources for young people with ADHD: *A mixed-methods systematic review and narrative synthesis*

European Child & Adolescent Psychiatry

Rebecca Gudka*, Elleie McGlynn, Katherine Lister, Naomi Shaw, Emma Pitchforth, Faraz Mughal, Blandine French, John Headly Ward, Tamsin Newlove-Delgado, Anna Price

*[r.gudka@exeter.ac.uk](mailto:r.gudka@exeter.ac.uk) - University of Exeter (Faculty of Health and Life Sciences), Exeter, UK.

# Appendix 2. Conference articles & thesis which were also identified

## Conference articles

Dibia V (2016) FOQUS: A Smartwatch Application for Individuals with ADHD and Mental Health Challenges. Proceedings of the 18th International ACM SIGACCESS Conference on Computers and Accessibility 311-312. <https://doi.org/10.1145/2982142.2982207>

Otto, S., Bemman, B., Brogaard Bertel, L., Knoche, H., Lassen Nørlem, H. (2022). Data-Driven User Profiling and Personalization in Tiimo: Towards Characterizing Time Management Behaviors of Neurodivergent Users of a Scheduling Application. In: Miesenberger, K., Kouroupetroglou, G., Mavrou, K., Manduchi, R., Covarrubias Rodriguez, M., Penáz, P. (eds) Computers Helping People with Special Needs. ICCHP-AAATE 2022. Lecture Notes in Computer Science, vol 13341. Springer, Cham, pp 442-50 <https://doi.org/10.1007/978-3-031-08648-9_51>

## Dissertations

Irvine MJD (2013) Outcome evaluation of a time management smartphone application: a pilot study. Dissertation, George Fox University

Nasri B (2017) New approach to the treatment of adhd through internet and smartphones - from impairment to improvement. Dissertation, Karolinska Institutet

Powell LA (2020) The design and evaluation of technology to self-manage long-term neurodisabilities across the lifespan. Dissertation, University of Sheffield
