# Supplementary material for: Digital health interventions with healthcare information and self-management resources for young people with ADHD: a mixed-methods systematic review and narrative synthesis
Source: Eur Child Adolesc Psychiatry. 2025 Mar 1;34(6):1817–35. doi: 10.1007/s00787-025-02676-y (PMC12198326; doi:10.1007/s00787-025-02676-y)
Supplement: Supplementary file 3 — Supplementary Material 3 [file 787_2025_2676_MOESM3_ESM.docx]

Digital health interventions including healthcare information and self-management resources for young people with ADHD: *A mixed-methods systematic review and narrative synthesis*

European Child & Adolescent Psychiatry

Rebecca Gudka*, Elleie McGlynn, Katherine Lister, Naomi Shaw, Emma Pitchforth, Faraz Mughal, Blandine French, John Headly Ward, Tamsin Newlove-Delgado, Anna Price

*[r.gudka@exeter.ac.uk](mailto:r.gudka@exeter.ac.uk) - University of Exeter (Faculty of Health and Life Sciences), Exeter, UK.

# Appendix 5. Glossary of intervention types

| **Intervention category** | **Definition** |
| --- | --- |
| Psychoeducation | Psychoeducation is education for people with ADHD regarding the symptoms they might be experiencing, which generally follow validated therapeutic manuals (e.g., Cognitive Behavioural Therapy) that may be adapted specifically towards ADHD. It is not simply 'providing information', but rather empowering training for patients targeted at promoting awareness, providing tools to manage, cope and live with ADHD, and changing behaviours and attitudes related to the condition. |
| Symptom monitoring | Symptom monitoring interventions are those which collect data from patients (either through self-reported questionnaires, or devices such as smartwatches which can detect movement and other physical changes) and provide summaries or visual presentations of symptoms. In some cases, these presentations are for the user themselves to monitor their symptoms over time, or to flag tools/strategies at the precise time a person is experiencing symptoms. In other cases, they can be used to help clinicians gain an understanding of the patients’ symptoms and how these may be affected by things such as medication use. |
| Practical tool | Practical tools vary in their nature, but generally facilitate self-management strategies by providing a template, prompt or activity. These are useful resources that support day-to-day tasks when managing a long-term condition. Practical tools generally rely less on providing information or changing behaviours/attitudes, rather offering stand-alone tools which may help to alleviate levels of impairment. |
| Healthcare & self-management information | Healthcare & self-management information are regarded as the least intensive intervention type for the purposes of this review. They generally provide information about ADHD, healthcare, self-management strategies and signposting to other resources, but do not follow therapeutic manuals, provide tools, or encourage behaviour change. They simply aim to raise awareness but do not deliver it in a modular manner or using strategies typically involved in psychoeducation. |
