# Supplementary material for: Digital health interventions with healthcare information and self-management resources for young people with ADHD: a mixed-methods systematic review and narrative synthesis
Source: Eur Child Adolesc Psychiatry. 2025 Mar 1;34(6):1817–35. doi: 10.1007/s00787-025-02676-y (PMC12198326; doi:10.1007/s00787-025-02676-y)
Supplement: Supplementary file 4 — Supplementary Material 4 [file 787_2025_2676_MOESM4_ESM.docx]

Digital health interventions including healthcare information and self-management resources for young people with ADHD: *A mixed-methods systematic review and narrative synthesis*

European Child & Adolescent Psychiatry

Rebecca Gudka*, Elleie McGlynn, Katherine Lister, Naomi Shaw, Emma Pitchforth, Faraz Mughal, Blandine French, John Headly Ward, Tamsin Newlove-Delgado, Anna Price

*[r.gudka@exeter.ac.uk](mailto:r.gudka@exeter.ac.uk) - University of Exeter (Faculty of Health and Life Sciences), Exeter, UK.

# Appendix 3. Reasons for exclusion of full-texts

| **Paper** | **Reason for exclude** |
| --- | --- |
| Acceptability and Feasibility of the Transfer of Face-to-Face Group Therapy to Online Group Chats in a Psychiatric Outpatient Setting During the COVID-19 Pandemic: Longitudinal Observational Study. Scholl J (2021) | Not digitally delivered |
| Acute and Long-Term Effects of an Internet-Based, Self-Help Comprehensive Behavioral Intervention for Children and Teens with Tic Disorders with Comorbid Attention Deficit Hyperactivity Disorder, or Obsessive Compulsive Disorder: A Reanalysis of Data from a Randomized Controlled Trial. Rachamim L (2021) | Not target age range/population |
| ADHD in adults: Psychoeducation with smartphone. [German]. Kessing R (2023) | No EngLang/High Income |
| ADHD: Is There an App for That? A Suitability Assessment of Apps for the Parents of Children and Young People With ADHD. Powell L (2017) | Not target age range/population |
| [ADHD: training brain performance with the computer helps]. [German]. (2012) | No EngLang/High Income |
| Attention Deficit Hyperactivity Disorder: Is There an App for That? Suitability Assessment of Apps for Children and Young People With ADHD. Powell L (2017) | Not target age range/population |
| Beliefs about attention-deficit/hyperactivity disorder and response to stereotypes: Youth postings in facebook groups. Gajaria A (2011) | Not for information/self-management |
| Brief Report: Pilot Study of a Novel Interactive Digital Treatment to Improve Cognitive Control in Children with Autism Spectrum Disorder and Co-occurring ADHD Symptoms. Yerys B (2019) | Not target age range/population |
| The caregivers' perspectives of burden before and after an internet-based intervention of young persons with ADHD or autism spectrum disorder. Soderqvist H (2017) | Not digitally delivered |
| Children with ADHD Show Improved Symptoms After Computer Program Training. (2011) | Not available |
| Cogmed Working Memory Training Presents Unique Implementation Challenges in Adults With ADHD. Marcelle E (2018) | Not for information/self-management |
| Cognitive assistive technology and professional support in everyday life for adults with ADHD. Lindstedt H (2013) | Not digitally delivered |
| Comparing the Transfer Effects of Three Neurocognitive Training Protocols in Children with Attention-Deficit/Hyperactivity Disorder: A Single-Case Experimental Design. Zhang D (2023) | Not target age range/population |
| Computer enabled neuroplasticity treatment: A clinical trial of a novel design for neurofeedback therapy in adult ADHD. Cowley B (2016) | Not for information/self-management |
| Computer-based attention training for treating a child with attention deficit/hyperactivity disorder: An adjunct to pharmacotherapy - A case report. Saha P (2015) | Not target age range/population |
| Developing an implementation model for adhd intervention in community clinics: Leveraging artificial intelligence and digital technology. Sibley M (2023) | Not target age range/population |
| Developing mHealth Remote Monitoring Technology for Attention Deficit Hyperactivity Disorder: A Qualitative Study Eliciting User Priorities and Needs. Simons L (2016) | Not digitally delivered |
| Development of an internet-based support and coaching model for adolescents and young adults with ADHD and autism spectrum disorders: A pilot study. Wentz E (2012) | Not digitally delivered |
| A double-blind randomized pilot trial comparing computerized cognitive exercises to Tetris in adolescents with attention-deficit/hyperactivity disorder. Bikic A (2017) | Not for information/self-management |
| Effectiveness of computer-based cognitive training, nutritional supplementations intervention and both combined on the improvement of attention, working memory, and behavioral symptoms of attention-deficit hyperactivity disorder. Barzegar M (2020) | Not for information/self-management |
| Effectiveness of online mindfulness-based intervention (Imbi) on inattention, hyperactivity-impulsivity, and executive functioning in college emerging adults with attention-deficit/hyperactivity disorder: A study protocol. Pheh K (2021) | No outcome measures/results |
| Effectiveness of web-based play therapy intervention in supporting the development of children with attention deficit/hyperactivity disorder. Budiyarti L (2023) | Not target age range/population |
| The Efficacy of Computerized Cognitive Training in Adults With ADHD: A Randomized Controlled Trial. Stern A (2016) | Not for information/self-management |
| Efficient Vocational Skills Training for People with Cognitive Disabilities: An Exploratory Study Comparing Computer-Assisted Instruction to One-on-One Tutoring. Larson J (2016) | Not for information/self-management |
| Empowerment of patients in online discussions about medicine use. van Berkel J (2015) | Not for information/self-management |
| An Evaluation Approach for the Performance of Dosing Regimens in Attention-Deficit/Hyperactivity Disorder Treatment. Bonnefois G (2017) | Not target age range/population |
| Evaluation of the Effectiveness of the FOCUS ADHD App in Monitoring Adults with Attention-Deficit/Hyperactivity Disorder. Carvalho L (2023) | Not target age range/population |
| Experiences of an internet-based support and coaching model for adolescents and young adults with ADHD and autism spectrum disorder-A qualitative study. Sehlin H (2018) | Not digitally delivered |
| Hazard perception skills of young drivers with Attention Deficit Hyperactivity Disorder (ADHD) can be improved with computer based driver training: An exploratory randomised controlled trial. Bruce C (2017) | Not for information/self-management |
| Health information-seeking behaviors about attention deficit hyperactivity disorder on the internet and the analysis of top ranking websites. Ersoy M (2015) | No outcome measures/results |
| Implementing a low-cost web-based clinical trial management system for community studies: a case study. Geyer J (2011) | Not target age range/population |
| Improvements of adolescent psychopathology after insomnia treatment: results from a randomized controlled trial over 1 year. de Bruin E (2018) | Not digitally delivered |
| Improving the quality of physical health monitoring in CAMHS for children and adolescents prescribed medication for ADHD. Oxley C (2018) | Not target age range/population |
| Individualised short-term therapy for adolescents impaired by attention-deficit/hyperactivity disorder despite previous routine care treatment (ESCAadol)-Study protocol of a randomised controlled trial within the consortium ESCAlife. Geissler J (2018) | No outcome measures/results |
| Innovative technological advancements to improve cognitive and social skills of students with neurodevelopmental disorders. Manta O (2020) | Not target age range/population |
| Leveraging Knowledge Graphs and Natural Language Processing for Automated Web Resource Labeling and Knowledge Mobilization in Neurodevelopmental Disorders: Development and Usability Study. Costello J (2023) | Not target age range/population |
| Living smart - a randomized controlled trial of a guided online course teaching adults with adhd or sub-clinical adhd to use smartphones to structure their everyday life. Moell B (2015) | Not digitally delivered |
| Mediated Online Awareness Among Adolescents With and Without ADHD: Using the Occupational Performance Experience Analysis (OPEA). Fisher O (2023) | No outcome measures/results |
| Online Cognitive Behavior Therapy for Two College Students With Attention-Deficit/Hyperactivity Disorder. Lopez-Pinar P (2023) | Not digitally delivered |
| Paging Dr. Google: Availability and Reliability of Online Evidence-Based Treatment Information about ADHD. King S (2021) | Not for information/self-management |
| Parents' perspectives on a smartwatch intervention for children with ADHD: Rapid deployment and feasibility evaluation of a pilot intervention to support distance learning during COVID-19. Cibrian F (2021) | Not target age range/population |
| Patient-centered design of an information management module for a personally controlled health record. Sox C (2010) | Not target age range/population |
| Proof-of-concept study of an at-home, engaging, digital intervention for pediatric ADHD. Davis N (2018) | Not target age range/population |
| The Quality of YouTube Videos as an Educational Resource for Attention-Deficit/Hyperactivity Disorder. Ward M (2020) | Not for information/self-management |
| A randomized controlled study of remote computerized cognitive, neurofeedback, and combined training in the treatment of children with attention-deficit/hyperactivity disorder. Luo X (2023) | Not target age range/population |
| A self-guided Internet-delivered intervention for adults with ADHD: A feasibility study. Nordby E (2021) | Not target age range/population |
| A self-guided Internet-delivered intervention for adults with ADHD: a protocol for a randomized controlled trial. Kenter R (2021) | No outcome measures/results |
| Smartphone-assisted psychoeducation in adult attention-deficit/hyperactivity disorder: A randomized controlled trial. Selaskowski B (2022) | Not digitally delivered |
| Smartphone-based contingency management for smoking cessation with smokers diagnosed with attention-deficit/hyperactivity disorder. Dan M (2016) | Not digitally delivered |
| A study protocol of a randomized controlled study of internet-based cognitive behavioral therapy for adult attention deficit hyperactivity disorder. Forsstrom D (2023) | No outcome measures/results |
| Sustained benefits of cognitive training in children with inattention, three-year follow-up. Jurigova B (2021) | Not target age range/population |
| Testing the efficacy of a smartphone application in improving medication adherence among children with ADHD. Weisman O (2017) | Not target age range/population |
| Testing the Efficacy of a Smartphone Application in Improving Medication Adherence, Among Children with ADHD. Weisman O (2018) | Not target age range/population |
| Use of training with BCI (Brain Computer Interface) in the management of impulsivity. Bonfiglio N (2020) | Not for information/self-management |
| Microtemporal Dynamics of Dietary Intake, Physical Activity, and Impulsivity in Adult Attention-Deficit/Hyperactivity Disorder: Ecological Momentary Assessment Study Within Nutritional Psychiatry. Ruf A (2023) | Not for information/self-management |
| Using a mobile phone-based application as an adjunct to facilitate oral hygiene practices in children with Attention Deficit Hyperactivity Disorder (ADHD). Gurnani H (2023) | Not target age range/population |
| A Digital Mental Health Support Program for Depression and Anxiety in Populations With Attention-Deficit/Hyperactivity Disorder: Feasibility and Usability Study. Tsirmpas C (2023) | Not digitally delivered |
| The understanding and managing adult ADHD programme: A qualitative evaluation of online psychoeducation with acceptance and commitment therapy for adults with ADHD. Seery C (2023) | Not digitally delivered |
| A brief office-based hazard perception intervention for drivers with ADHD symptoms. Poulsen A (2010) | Not for information/self-management |
| Can reduce - the effects of chat-counseling and web-based self-help, web-based self-help alone and a waiting list control program on cannabis use in problematic cannabis users: a randomized controlled trial. Schaub M (2013) | No outcome measures/results |
| Psychoeducation for adults with attention deficit hyperactivity disorder vs. cognitive behavioral group therapy: a randomized controlled pilot study. Vidal R (2013) | Not available |
| Internet-Based Support and Coaching With Complementary Clinic Visits for Young People With Attention-Deficit/Hyperactivity Disorder and Autism: Controlled Feasibility Study. Sehlin H (2020) | Not digitally delivered |
