# Supplementary material for: Digital health interventions with healthcare information and self-management resources for young people with ADHD: a mixed-methods systematic review and narrative synthesis
Source: Eur Child Adolesc Psychiatry. 2025 Mar 1;34(6):1817–35. doi: 10.1007/s00787-025-02676-y (PMC12198326; doi:10.1007/s00787-025-02676-y)
Supplement: Supplementary file 5 — Supplementary Material 5 [file 787_2025_2676_MOESM5_ESM.docx]

Digital health interventions including healthcare information and self-management resources for young people with ADHD: *A mixed-methods systematic review and narrative synthesis*

European Child & Adolescent Psychiatry

Rebecca Gudka*, Elleie McGlynn, Katherine Lister, Naomi Shaw, Emma Pitchforth, Faraz Mughal, Blandine French, John Headly Ward, Tamsin Newlove-Delgado, Anna Price

*[r.gudka@exeter.ac.uk](mailto:r.gudka@exeter.ac.uk) - University of Exeter (Faculty of Health and Life Sciences), Exeter, UK.

# Appendix 4. Descriptions of interventions, with relevant outcomes

| **Intervention name [studies]** | **Mode of delivery** | **Brief description** | **Relevant outcome domains** |
| --- | --- | --- | --- |
| **Psychoeducation** | | | |
| **CANReduce** Ahlers (2022, Switzerland) | Web-based | Automated web-based self-help tool to reduce cannabis consumption in problematic cannabis users. Consists of a dashboard, consumption diary, and eight psychoeducation modules. | ADHD symptoms (ASRS)  Other health-related outcome (Depression (CES-D), Anxiety (GAD))  Cannabis use disorder identification test (CUDIT & SDS & number of days cannabis consumed) |
| **InFlow** Knouse (2022, USA) | Mobile app | Science-based digital CBT-based psychoeducation app guiding users to implement ADHD-relevant behavioural/cognitive skills in day-to-day life. Allows users to track progress and access online community of like-minded users. | Usability (13-item scale)  Feasibility/acceptability (App use data)  ADHD symptoms (BAARS & BFIS) |
| **MyADHD** Flobak (2021) Nordby (2022, Norway) | Web-based | Short-term, structured self-guided intervention with modified elements from CBT, DBT and GMT to target specific challenges experienced by adults with ADHD. Seven training modules released weekly. Goal of intervention to improve daily-life functioning; provide stress-reducing strategies; reduce inattention; and improve quality of life. Each module includes multimedia psychoeducation and instructions for specific techniques. Automatic reminders are sent when participants do not log on or complete a module. Additional SMS reminders sent to some participants in Nordby 2022. | Intervention engagement (module completion rate, login number, minutes spent online & use of coping strategies)  Usability (Module satisfaction rating) |
| **MyADHD** Kenter (2022, Norway) |  |  | Usability (think-aloud interview data) |
| **MyADHD** Kenter (2023, Norway) |  |  | ADHD symptoms (ASRS)  Other health related outcome (QoL (AAQoL), Stress (PSS))  Feasibility/acceptability (Client satisfaction questionnaire CSQ-8) |
| **Tokadi** Jang (2021, Korea) | Mobile app (Chatbot) | Conversational Chatbot mobile app with format similar to text messaging application. Provides user with psychoeducation & self-help strategies. As well as empathetic responding with dialogue skills. Mascot also provides emojis according to the situation so that emotional rapport can be established. | ADHD symptoms (CAARS)  Other health related outcomes (Depression (QUIDS-SR), Anxiety (SAS), Stress (PSS))  Feasibility/acceptability (rating scales)  Usability (open ended questions  Intervention engagement (app use data) |
| **Chatbot based psychoeducation** Selasowski (2023, Germany) | Mobile app (Chatbot) | Psychoeducation content consisted of eight modules with a comprehensive summary of the content, assignments and a content quiz. Chatbot interactively presents content based on user input. | ADHD symptoms (ADHS-SB) (IDA-R)  Quality of life (WHOQOL), Depression, Anxiety and Stress (DASS-21)  Intervention engagement (number of correct/missing quiz questions) |
| **Internet-based intervention** Shelton (2022, USA) | Web-based | Proposed intervention has treatment content algorithmically tailored to individuals based on ADHD symptoms and related impairment. Seven modules, the first compulsory for all users, the rest only presented to users who answered certain questions on ADHD and impairment questionnaires | Feasibility/acceptability (Treatment credibility & acceptance scale (CEQ))  Preference & relevance (Open endd questions) |
| **Internet-based CBT** Nasri (2023, Sweden) | Web-based | Combination of CBT and DBT methods. Text and media-based self-help material divided into modules with educative material; descriptions, rationales, and instructions for therapeutic techniques; quick, often self-reflective, exercises; and more time demanding homework assignments. | ADHD symptoms (ASRS)  Other health related outcomes (ADHD related life quality (AaQoL), Depression symptoms (MADRS-S), Emotion regulation (DERS), Perceived stress (PSS-4), Work ability (WAI-I), Life satisfaction (SWLS), Functional impairment (SDS), General health % Life quality (EQ-5D Index), Self-rated health (EQ5D VAS))  Feasibility/usability (TCS) |
| **InFocus** Petterson (2017, Sweden) | Web-based | Both the iCBT-G and iCBT-S groups followed the iCBT program In Focus. Based on therapeutic methods from CBT and focuses mainly on teaching compensatory techniques to handle ADHD-related difficulties, such as behavior analyses, time management, problem solving, and organization and planning but also on how to handle dysfunctional thinking (cognitive restructuring) and emotional distress (mindfulness and acceptance techniques). 9 modules - each module has information component + excersise component with theraputic technique. iCBT-S was self-guided but with optional + non-scheduled support function with therapist to ask questions | ADHD symptoms (CSS)  Other health-related outcomes (Depression (BDI), Anxiety (BAI), Quality of life (ADHD impact module), Occupational performance (COPM))  Feasibility/acceptability (Usage data and retention rate) |
| **Healthcare/self-management information** | | | |
| **IUEVO** Wright (2023, USA) | Website | Iuveo is a website aiming to empower teenagers to take control of their health by providing written information and videos on common conditions, including ADHD and stimulant use, for their age group. | Information preferences (advisory board meetings) |
| **NR** Luiu (2018, Switzerland) | Mobile app | App based on Precaution Adoption Process Model which delivers tailored messages, advices, information, exercises and feedbacks. | Perceived helpfulness, ergonomics & willingness to use (semi-structured interview) |
| **Symptom monitoring** | | | |
| **StopWatch** Leikauf (2019, USA) | Smart (Apple) watch & mobile | An application developed for a wearable device (Apple Watch) that was designed to track movement and provide visual and haptic feedback for ADHD. | ADHD symptoms  Intervention engagement  Feasibility/acceptability |
| **FitBit Flex app** Schoenfelder (2017, USA) | Smart (Fitbit Flex) watch & mobile | An application developed for a wearable device (Fitbit Flex) which collects accelerometer data as proxy estimate of physical activity including steps, energy expended, and distance travelled. The Fitbit mHealth mobile app provides graphs of the data and feedback toward personalized goal attainment. Participants joined a private, invitation-only FB group created for the study to interact with other participants and receive study information and encouragement, with digital badges for meeting goals, social interactions or making improvement toward goals. | ADHD symptoms  Other health-related outcome (physical activity)  Intervention engagement  Feasibility/acceptability |
| **SMS treatment optimisation** Surman (2022, USA) | SMS | Provides internet and SMS-based tool for participants to report severity of ADHD-related traits they thought would be sensitive to presence of ADHD medication to monitor symptoms to assist treatment optimisation. Participants were notified to complete a survey on 20 different occasions over 10 days.  Each survey episode included four questions: (1) a question about whether they took medication that day, (2) the pre-determined items from both the WFIRS-S, and (3) the ASRS, as well as (4) an additional item from the ASRS they did not choose. | ADHD symptoms  Usability |
| **Ecological Momentary Assessment** Kennedy (2022, USA) | Web-based & SMS | SMS prompts sent to participants 4 times daily for 17 days to remind participants to fill out EMA, ecological momentary assessment, measures (ADHD symptom severity). EMA was used to allow participants to self-monitor day-to-day symptoms based around inattentiveness, impulsivity and hyperactivity. | ADHD symptoms  Medication adherence  Intervention engagement z |
| **Management tool** | | | |
| **SMS Medication Reminders** Biederman (2019, USA) | SMS | Text messaging technology tailored to address four key areas in ADHD: 1) reminders to take the stimulants as prescribed; 2) reminders to renew the prescriptions in a timely fashion; 3) educational reminders about ADHD and its treatment; and 4) useful tips about time management and organizational needs aimed at more efficiently handling activities of daily living. | Medication adherence (whether patients had timely refills for prescriptions within certain number of days from index date) |
| **SMS Medication Reminders** Biederman (2020, USA) |  | Same intervention in primary care setting |  |
